# Negative Magnetization Phenomena in A‑Site Columnar-Ordered Quadruple Perovskites Ce_2_MnM(Mn_2_Sb_2_)O_12_ with M = Mn and Zn

**DOI:** 10.1021/acs.inorgchem.5c00653

**Published:** 2025-05-16

**Authors:** Xuan Liang, Kazunari Yamaura, Alexei A. Belik

**Affiliations:** † Research Center for Materials Nanoarchitectonics (MANA), 52747 National Institute for Materials Science (NIMS), Namiki 1-1, Tsukuba, Ibaraki 305-0044, Japan; ‡ Graduate School of Chemical Sciences and Engineering, Hokkaido University, North 10 West 8, Kita-ku, Sapporo, Hokkaido 060-0810, Japan

## Abstract

A phenomenon of magnetization reversal in response to an applied magnetic field is very common and forms the basis of magnetic memories. In contrast, the phenomenon of magnetization reversal in response to a temperature change is rarer. In this work, we demonstrated a pronounced negative magnetization effect (NME) during field-cooled measurements in small magnetic fields in members of the A-site columnar-ordered quadruple perovskites, Ce_2_MnM­(Mn_2_Sb_2_)­O_12_ with M = Mn and Zn, which were prepared by a high-pressure, high-temperature method at about 6 GPa and about 1600 K. Their crystal structures at room temperature were investigated with synchrotron X-ray powder diffraction data. Both compounds crystallize in space group *P*4_2_/*n* (No. 86) with full rock-salt ordering of Mn and Sb at the B sites. Lattice parameters are *a* = 7.84545(1) and *c* = 7.95529(2) Å for M = Mn and *a* = 7.81270(1) and *c* = 7.94100(1) Å for M = Zn. The bond-valence sum analysis and the charge balance suggest that cerium is present in the oxidation state of +3. They show one magnetic transition at *T*_C_ = 52 K with the compensation point near 20 K for M = Mn, and at *T*_C_ = 34 K with the compensation point near 29 K for M = Zn. A robust, intrinsic NME was also observed on zero-field-cooled curves when measured in small magnetic fields. The NME could originate from its ferrimagnetic structures.

## Graphical abstract

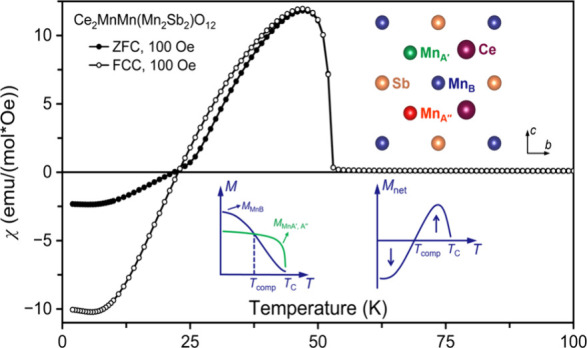

## 1 Introduction

The negative magnetization effect (NME) or phenomenon, also known as magnetization reversal, refers to an uncommon magnetic behavior in which the magnetization of a material aligns antiparallel to an applied magnetic field. Specifically, NME describes the crossover of direct-current magnetization from positive to negative under a positive magnetic field as temperature decreases.^1–3^ This effect deviates from conventional magnetism, where magnetization typically aligns with the applied field and arises due to competing magnetic interactions that disrupt conventional ordering. The origins of this behavior are linked to complex interplays between spin dynamics, magnetic anisotropy, crystal symmetry, and the interactions among magnetic ions.^4–8^

Research into NME has garnered considerable interest due to its potential to reveal new magnetic states and provide deeper insights into the fundamental physics governing magnetic materials.^1–3^ Moreover, the dual tunability of magnetization by both magnetic fields and temperature offers promising avenues for future applications in advanced magnetic storage technologies, spintronic devices, and other areas where precise control of magnetic states is critical.^3,9–11^

This phenomenon has been observed and studied in various perovskite materials. In ABO_3_ perovskites, magnetic interactions are primarily dictated by the arrangement of magnetic ions at the A and B sites, where competition between different exchange pathways can lead to magnetization reversal.^12^ For instance, in (Tm_1–*x*_Mn_*x*_)­MnO_3_ solid solutions, the ordered Tm^3+^ moments significantly increase at low temperatures, overpowering the saturated magnetic Mn moments at the B site. This results in magnetization reversal with a compensation temperature (*T*_comp_) of around 15 K in the *x* = 0.2 and 0.3 samples under small magnetic fields.^13^ Similarly, in rare-earth-based manganite materials such as NdMnO_3_ and Gd_0.5_Sr_0.5_MnO_3_, negative magnetization arises from the negative exchange interaction between the rare-earth ions and Mn sublattices.^14,15^

In double perovskites (A_2_BB′O_6_), the coexistence of two distinct magnetic ions at the B and B′ sites introduces additional complexity to the magnetic interactions.^16^ For example, in A_2_CoMnO_6_ double perovskites, where A is a rare-earth element, the competition between ferromagnetic (FM) superexchange interaction of Jahn–Teller (JT)-active Co^2+^ and JT-inactive Mn^4+^ and supplementary antiferromagnetic (AFM) interactions arising from the antisite disorder caused by the interchange of crystallographic positions between Co and Mn can result in spin frustration and the emergence of NME.^17,18^

Quadruple perovskites also present promising candidates for NME due to the inclusion of additional magnetic sites, which enhances inter-sublattice interactions. Quadruple perovskites with the general composition AA′_3_B_4_O_12_ feature a 12-fold coordinated A site and a square-planar coordinated A′ site, where the A′ site is usually occupied by Cu^2+^, Fe^2+^, Mn^2+^, or Mn^3+^.^19–23^ Strong interactions between the 3d transition metals at the A′ and B sites can lead to simultaneous magnetic ordering across these cations.^19,20^ However, to the best of our knowledge, NME was not observed in AA′_3_B_4_O_12_ perovskites. Quadruple perovskites A_2_A′A″B_4_O_12_ have original columnar-type arrangements of A cations with one column containing A positions and another column containing alternating A′ and A″ positions (the so-called A-site columnar-ordered quadruple perovskite). The presence of magnetic cations in new arrangements and unusual coordination environments could lead to complex interactions among magnetic cations located in the A′, A″, and B sites resulting in different magnetic ground states and spin-reorientation transitions.^21,22^

It was recently found that the majority of A_2_A′A″B_4_O_12_ perovskites have ferrimagnetic (FIM) structures; however, no significant NME was observed.^21,23^ This outcome was unexpected, as FIM systems are especially prone to exhibiting NME.^3^ In FIM systems, magnetic moments of different ions are aligned in opposite directions, similar to AFM systems, but with unequal magnitudes.^24,25^ This imbalance results in net magnetization. Under certain conditions, such as low temperatures or specific external magnetic fields, the antiparallel moments in FIM materials can become dominant, leading to a reversal of overall magnetization. This makes FIM materials highly susceptible to magnetization reversal, as the delicate competition between sublattice magnetizations can easily shift, resulting in a crossover from positive to negative magnetization.^26–28^ For example, neutron diffraction measurements reveal that the origin of negative magnetization in (Tm_0.7_Mn_0.3_)­MnO_3_ lies in its FIM structure and the differing temperature dependences of the sublattice magnetizations.^13^

In this work, we report the observation of the negative magnetization effect (NME) in A-site columnar-ordered quadruple perovskites, Ce_2_MnM­(Mn_2_Sb_2_)­O_12_ (M = Mn, Zn). These compounds crystallize in space group *P*4_2_/*n* (No. 86) with full Mn and Sb rock-salt ordering at the B sites. They undergo one magnetic transition near *T*_C_ = 52 K for M = Mn and *T*_C_ = 34 K for M = Zn. During field-cooled measurements in small magnetic fields, both materials display pronounced NME, which is also evident in zero-field-cooled curves under similar conditions. The observed NME is likely attributable to their FIM structures, highlighting the influence of complex magnetic interactions in these materials and the critical role of multimoment vector arrangements and anisotropy coupling.

## 2 Experimental Section

Ce_2_MnM­(Mn_2_Sb_2_)­O_12_ samples with M = Mn and Zn were prepared from stoichiometric mixtures of CeO_2_, MnO or ZnO, Mn_3_O_4_, and Sb_2_O_3_ (all 99.9%) at about 6 GPa and about 1600 K for 2 h in Au capsules by a high-pressure, high-temperature method. After being annealed at 1600 K, the samples were cooled to room temperature (RT) by turning off the heating current, and the pressure was slowly released. Before use, CeO_2_ was dried in air at 1270 K for 1 h and the other oxides were dried in air at 390 K for 4 h. No uncommon hazards were noted.

X-ray powder diffraction (XRPD) data were collected on a RIGAKU MiniFlex600 diffractometer using Cu Kα radiation at RT (2θ range of 5–100° with a step of 0.02° and a scan speed of 3°/min). Synchrotron XRPD data were collected on the BL02B2 beamline of SPring-8 at RT between 1.95 and 71.25° at a 0.006° interval in 2θ with the wavelength of λ = 0.61974 Å.^29^ The data from 5° (for M = Mn) and 4° (for M = Zn) were used in the refinements, as no experimental reflections were observed below these values. The samples were placed into Lindemann glass capillary tubes (inner diameter: 0.2 mm), which were rotated during measurements. The Rietveld analysis of all XRPD data was performed using the *RIETAN-2000* program.^30^

Magnetic measurements were performed on a SQUID magnetometer (Quantum Design, MPMS3) between 2 and 300 K in an applied magnetic field of 100 and 10 kOe under both zero-field-cooled (ZFC) and field-cooled on cooling (FCC) conditions. Additional magnetic measurements were also performed between 2 and 70 K in different applied fields under ZFC and FCC conditions. The inverse magnetic susceptibilities (*χ* ^–1^) were fit by the Curie–Weiss equation

$$\chi \left(T\right)=\mu_{eff}^{2}N{\left(3k_{B}\left(T-\Theta \right)\right)}^{-1}$$

where μ_eff_ is the effective magnetic moment, *N* is Avogadro’s number, *k*_B_ is Boltzmann’s constant, and Θ is the Curie–Weiss temperature. For fitting, we used the FCC data at *H* = 10 kOe between 200 and 295 K. Isothermal magnetization measurements were performed between −70 and 70 kOe at different temperatures (5, 10, 20, 30, 40, 50, and 60 K). Specific heat was measured on a Quantum Design PPMS-9T instrument on cooling at magnetic fields of 0 Oe and 90 kOe.

## 3 Results and Discussion

### 3.1 Crystal Structure Determination and Description

The synchrotron XRPD patterns of Ce_2_MnM­(Mn_2_Sb_2_)­O_12_ samples with M = Mn and Zn are shown in Figure 1a,b (with a zoom-in of the low-2θ region in Figure S1). Both samples crystallize in a tetragonal system with space group *P*4_2_/*n*. The M = Mn sample contained 3.6 wt % CeO_2_, 0.2 wt % cubic-pyrochlore (space group *Fd*3̅*m*, *a* = 10.2633 Å, which could be Sb_2_O_4+*x*_), and 2.9 wt % La_3_Mn_2_Sb_3_O_14_-type pyrochlore^31^ (space group *R*3̅*m*, *a* = 7.4262 Å and *c* = 17.6394 Å) impurities while the M = Zn sample contained 4.2 wt % CeO_2_, 7.9 wt % La_3_Mn_2_Sb_3_O_14_-type pyrochlore^31^ (space group *R*3̅*m*, *a* = 7.4428 Å and *c* = 17.6257 Å), and 3.7 wt % Na_5_Co_15.5_Te_6_O_36_-type^32^ (space group *P*6_3_/*m*, *a* = 9.6229 Å and *c* = 9.3509 Å) impurities. The lattice parameters of Ce_2_MnMn­(Mn_2_Sb_2_)­O_12_ were *a* = 7.84545(1) Å and *c* = 7.95529(2) Å, and those of Ce_2_MnZn­(Mn_2_Sb_2_)­O_12_ were *a* = 7.81270(1) Å and *c* = 7.94100(1) Å. The rare-earth stability range (at certain synthesis conditions) of A-site columnar ordered quadruple perovskites usually strongly depends on the occupation of the A′, A″, and B sites and is a subject to considerable restrictions;^21,23^ for example, R_2_MnMnMn_4_O_12_ is stable for R = Gd–Er, Y,^33^ R_2_MnMn­(MnTi_3_)­O_12_ is stable for R = Nd–Gd,^34^ and NaRMn_2_Ti_4_O_12_ is stable for Sm, Eu, Gd, Dy, Ho, and Y.^35^ It was found that R_2_MnMn­(Mn_2_Sb_2_)­O_12_ are stable for R = La, Pr, Nd, and Sm;^36–38^ however, R = Ce was omitted from the investigation in ref 36. As R_2_MnZn­(Mn_2_Sb_2_)­O_12_ has a different combination of A′, A″, and B cations, we also preliminarily investigated the rare-earth stability range of R_2_MnZn­(Mn_2_Sb_2_)­O_12_ and prepared such compounds with R = Nd, Eu, Dy, and Yb. It was found that R_2_MnZn­(Mn_2_Sb_2_)­O_12_ can be stabilized for R = Nd and Eu (unpublished data) in the A-site columnar-ordered perovskite structure. On the other hand, samples with R = Dy and Yb crystallized in a double-perovskite structure (space group *P*2_1_/*n*) with a statistical distribution of R^3+^, Mn^2+^, and Zn^2+^ cations at one A site. Stabilization of Ce^3+^ (see below for confirmation) in the A site of quadruple perovskites is uncommon^39^ because the Ce^4+^ oxidation state is the most stable form in comparison with R_2_O_3_ (R = La, Nd, Sm, Eu, Gd, Dy–Lu, and Y) with the R^3+^ oxidation state, and Ce_2_O_3_ is easily oxidized.^40^ We also tried to synthesize Ce_2_MnMn­(Mn_2_Sb_2_)­O_12_ and Ce_2_MnZn­(Mn_2_Sb_2_)­O_12_ at a higher temperature of 1800 K (at 6 GPa in Pt capsules for 2 h); however, the samples contained CeO_2_ and Sb_2_O_4+*x*_ (a pyrochlore-type structure) as the main phases, suggesting that the samples decomposed (Figure S2).

**Figure 1:**
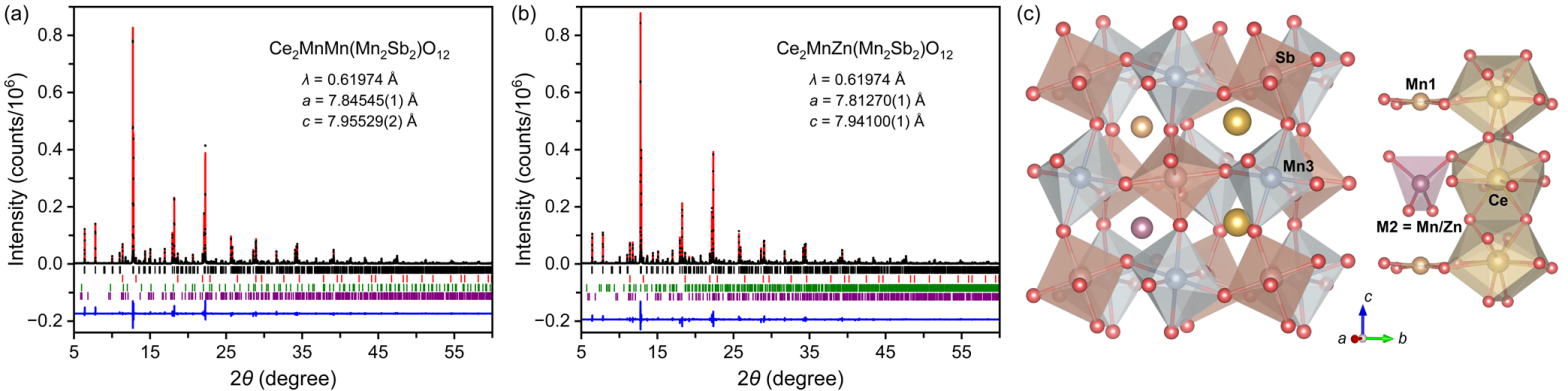
Fragments of experimental (black circles), calculated (red line), and difference (blue line) synchrotron X-ray powder diffraction patterns of (a) Ce_2_MnMn­(Mn_2_Sb_2_)­O_12_ and (b) Ce_2_MnZn­(Mn_2_Sb_2_)­O_12_ at *T* = 295 K. On the panel (a), the tick marks show possible Bragg reflection positions of the main perovskite phase (the first black row), CeO_2_ impurity (the second red row), cubic pyrochlore impurity (the third green row), and La_3_Mn_2_Sb_3_O_14_-related pyrochlore impurity (the fourth purple row). On the panel (b), the tick marks show possible Bragg reflection positions of the main perovskite phase (the first black row), CeO_2_ impurity (the second red row), Na_5_Co_15.5_Te_6_O_36_-type impurity (the third green row), and La_3_Mn_2_Sb_3_O_14_-related pyrochlore impurity (the fourth purple row). (c) Crystal structure of Ce_2_MnM­(Mn_2_Sb_2_)­O_12_ with M = Mn and Zn in a polyhedral presentation. The rock-salt-type arrangement of Mn3O_6_ and SbO_6_ octahedra is shown on the left. The columnar-type arrangement of CeO_10_ polyhedra, Mn1O_4_ square planar units, and (Mn/Zn)­O_4_ = M2O_4_ tetrahedra is shown on the right.

Structure parameters of Nd_2_MnMn­(Mn_4–*x*_Sb_*x*_)­O_12_ ^36,38^ were used as the initial model for the crystal structure refinements of Ce_2_MnM­(Mn_2_Sb_2_)­O_12_ with M = Mn and Zn. We initially assume that all cations were distributed in ideal sites: Ce^3+^ at the 10-fold-coordinated A site, Mn^2+^ at the square-planar A′ site (Mn1), and Mn^2+^/Zn^2+^ at the tetrahedral A″ site (M2). For the sample Ce_2_MnZn­(Mn_2_Sb_2_)­O_12_, Mn^2+^ cations (23 electrons) and Zn^2+^ cations (28 electrons) differ by 5 electrons or around 20%. This difference is enough to identify them with high-quality synchrotron powder X-ray powder diffraction.

Refinements of the occupation factors (*g*) of the Ce site (simultaneously with all atomic displacement parameters (and other parameters), but with fixed *g* parameters for other sites) gave the following values: *g*(Ce) = 0.960(2) for M = Mn and *g*(Ce) = 0.991(2) for M = Zn. Such deviations can be absorbed by reasonable atomic displacement parameters (e.g., *B*(Ce) = 0.693(10) Å^2^ with *g*(Ce) = 1 for M = Mn). Nevertheless, we assumed the presence of an antisite disorder when *g* values deviated from 1 by more than 3%. Therefore, the presence of Mn at the Ce site for M = Mn was assumed in the final model, and we refined the cation distribution with a constraint on the full site occupation; e.g., *g*(Ce) + *g*(Mn) = 1. The occupation factor of the Ce site was fixed at 1 for M = Zn in the final model. Refined *g* parameters (simultaneously) for the Sb and Mn3 (or M3) sites (under the same conditions as above) were *g*(Sb) = 1.001(3) and *g*(Mn3) = 0.979(4) for M = Mn and *g*(Sb) = 1.022(3) and *g*(Mn3) = 1.050(5) for M = Zn. Therefore, the *g*(Sb) values were fixed at 1 in the final models. Refined *g* parameters for the Mn1 site (under the same conditions as above) were *g*(Mn1) = 0.972(3) for M = Mn and *g*(Mn1) = 1.018(7) for M = Zn. Therefore, the *g*(Mn1) values were fixed at 1 in the final models. Refined *g* parameters for the M2 site (under the same conditions as above) were *g*(Mn2) = 0.974(6) for M = Mn and *g*(Zn2) = 0.922(6) for M = Zn. Based on these values, *g*(Mn2) was fixed at 1 for M = Mn. On the other hand, *g*(Zn2) and *g*(Mn3) values for M = Zn could suggest some antisite disorder of Zn^2+^ and Mn^2+^ between the M2 and Mn3 sites. Therefore, we refined the cation distribution between these two sites with constraints on the full site occupation and the total chemical composition. The final crystallographic and structure parameters are presented in Tables 1 and 2. We note that when only Mn was assumed at the M2 site of Ce_2_MnZn­(Mn_2_Sb_2_)­O_12_ its refined occupation factor was 1.144(7).

**Table 1:** Crystallographic Parameters and Structure Refinement Details of Ce_2_MnM­(Mn_2_Sb_2_)­O_12_ with M = Mn and Zn^a^

	M	
	Mn	Zn
*a* (Å)	7.84545(1)	7.81270(1)
*c* (Å)	7.95529(2)	7.94100(1)
*V* (Å^3^)	489.657(1)	484.705(1)
molecular weight (g/mol)	935.5048	945.9468
*R*_wp_ (%)	8.49	9.08
*R*_p_ (%)	6.42	6.88
*R*_I_ (%)	3.21	3.50
*R*_F_ (%)	1.92	2.23

a Synchrotron X-ray powder diffraction (λ = 0.61974 Å). *T* = 295 K. 2θ range used in the refinement: 5–71.25° for M = Mn sample, 4–71.25° for M = Zn sample. Crystal system: tetragonal. Space group: *P*4_2_/*n* (No. 86, cell choice 2), *Z* = 2.

**Table 2:** Structure Parameters of Ce_2_MnM­(Mn_2_Sb_2_)­O_12_ with M = Mn and Zn at 295 K from Synchrotron X-ray Powder Diffraction Data^a^

M	Site	WP	*x*	*y*	*z*	*B*_iso_ (Å^2^)
Mn	Ce	4*e*	0.25	0.75	0.77610(6)	0.580(11)
	Mn1-SQ	2*b*	0.25	0.25	0.75	1.17(9)
	Mn2-T	2a	0.75	0.75	0.75	0.61(8)
	Mn3-Oc	4*c*	0	0.5	0.5	0.65(2)
	Sb-Oc	4*d*	0	0	0.5	0.375(9)
	O1	8*g*	–0.0461(9)	0.5739(9)	0.2345(7)	0.52(11)
	O2	8*g*	–0.2348(12)	–0.0430(7)	0.5831(6)	0.90(12)
	O3	8*g*	–0.2587(10)	0.0671(6)	–0.0319(6)	0.51(9)
Zn	Ce	4*e*	0.25	0.75	0.77499(7)	0.647(10)
	Mn1-SQ	2*b*	0.25	0.25	0.75	0.63(10)
	M2-T	2a	0.75	0.75	0.75	1.00(11)
	M3-Oc	4*c*	0	0.5	0.5	0.69(3)
	Sb-Oc	4*d*	0	0	0.5	0.364(11)
	O1	8*g*	–0.0532(15)	0.5701(15)	0.2327(9)	1.10(15)
	O2	8*g*	–0.2291(13)	–0.0480(8)	0.5889(8)	0.85(14)
	O3	8*g*	–0.2591(13)	0.0701(8)	–0.0365(8)	1.28(14)

a WP is Wyckoff position. For M = Mn sample, *g*(Ce) = 0.928(3)Ce + 0.072Mn and *g*(Mn1-SQ) = *g*(Mn2-T) = *g*(Mn3-Oc) = *g*(Sb-Oc) = *g*(O1) = *g*(O2) = *g*(O3) = 1, where *g* is the occupation factor. For M = Zn sample, *g*(Ce) = *g*(Mn1-SQ) = *g*(Sb-Oc) = *g*(O1) = *g*(O2) = *g*(O3) = 1, *g*(M2-T) = 0.65(2)Zn + 0.35Mn, and *g*(M3-Oc) = 0.823Mn + 0.177Zn. Abbreviations: SQ, square-planar (site); T, tetrahedral (site); Oc: octahedral (site).

The crystal structure of Ce_2_MnM­(Mn_2_Sb_2_)­O_12_ samples with M = Mn and Zn is illustrated in Figure 1c. In this A-site columnar-ordered quadruple perovskite structure with the *P*4_2_/*n* space group (No. 86), the Mn (Mn3) and Sb atoms each are located at an octahedral center, and the Mn3/SbO_6_ octahedra are alternately corner-connected in a rock-salt arrangement.^41^ It has two in-phase and one out-of-phase tilts of the Mn3/SbO_6_ octahedra (written *a* ^+^ *a* ^+^ *c* ^–^ in the Glazer notation^42^) which creates 10 and 4 coordination numbers around the three A sites.^21^ As shown on the right of Figure 1c, the 10-coordinated A-site CeO_10_ polyhedra are connected through edges and form columns along the *c* axis. The 4-coordinated A′-site Mn1O_4_ squares and 4-coordinated A″-site M2O_4_ tetrahedra (M2 = Mn/Zn) are separated from each other but connected with A-site CeO_10_ polyhedra through edges and corners, respectively.

Table 3 shows the bond lengths, bond angles, bond-valence sum (BVS),^43,44^ and distortion parameters. The BVS values for the Ce site (+2.82 and +2.91)^44^ confirm the +3 oxidation state. There are two quite long Ce–O1 bond lengths (3.004(9) and 2.936(15) Å) in the *P*4_2_/*n* structure of Ce_2_MnM­(Mn_2_Sb_2_)­O_12_ samples in agreement with other R_2_MnMn­(Mn_2_Sb_2_)­O_12_ samples,^36–38^ but in contrast with the *P*4_2_/*nmc* structure of other related compounds without B-site double ordering.^37,38^ The BVS value of +1.77 for the Mn1 site in the M = Mn sample is consistent with the slightly elongated Mn1–O1 bond length of 2.117(5) Å (compared with the M = Zn sample, BVS = +1.90, and *l*(Mn1–O1) = 2.088(6) Å). Reduced BVS values for the square-planar site are often observed in such perovskites. For the M2 and M3 sites, the BVS values (+1.86 and +2.11 for the M = Mn sample; +1.74 and +1.99 for the M = Zn sample) also supported the oxidation state +2. The M2–O2 bond lengths differ noticeably for M = Mn (2.101(5) Å) and M = Zn (2.038(6) Å), reflecting different ionic radii of Mn^2+^ (*r*_IV_ = 0.66 Å) and Zn^2+^ (*r*_IV_ = 0.60 Å).^45^ The Zn–O bond lengths in Ce_2_MnZn­(Mn_2_Sb_2_)­O_12_ were close, for example, to those of Dy_2_MnZn­(Mn_3_Ti)­O_12_ and Dy_2_MnZn­(Mn_2_Ti_2_)­O_12_.^46^ The BVS values at the Sb site (+5.35 and +5.55) were slightly higher than anticipated, implying that Sb^5+^ cations tend to be overbonded. This conclusion is further supported by the relatively short Sb–O bond lengths (1.981(8)–1.987(5) Å for the M = Mn sample and 1.960(10)–1.982(10) Å for the M = Zn sample). Interestingly, similar trends in the BVS values of Sb^5+^ and bond lengths have been observed in other A-site columnar-ordered quadruple perovskites where Sb occupies the B-site.^36–38^ The resultant charge distribution is Ce^3+^_2_Mn^2+^M^2+^(Mn^2+^_2_Sb^5+^_2_)­O_12_, while Ce usually takes the +4 oxidation state in other A-site-ordered quadruple perovskites, such as CeCu_3_Fe_4_O_12_,^47^ CeCu_3_Mn_4_O_12_,^48^ and CeCu_3_Cr_4_O_12_.^49^

**Table 3:** Bond Lengths (in Å), Bond Angles (in deg), Bond-Valence Sum (BVS), and Distortion Parameters of MnO_6_ and SbO_6_ (Δ) in Ce_2_MnM­(Mn_2_Sb_2_)­O_12_ with M = Mn and Zn at *T* = 295 K from Synchrotron X-ray Powder Diffraction Data^a^

	M	
	Mn	Zn
Ce–O1 × 2	2.723(9)	2.775(15)
Ce–O1 × 2	3.004(9)	2.936(15)
Ce–O2 × 2	2.560(5)	2.572(6)
Ce–O3 × 2	2.417(5)	2.359(6)
Ce–O3 × 2	2.491(5)	2.508(6)
BVS(Ce^3+^)	+2.82	+2.91
Mn1–O1 × 4	2.117(5)	2.088(6)
Mn1–O2 × 4	3.110(5)	3.124(6)
BVS(Mn1^2+^)	+1.77	+1.90
M2–O2 × 4	2.101(5)	2.038(6)
M2–O3 × 4	3.034(5)	3.022(6)
BVS(M2^2+^)	+1.86	+1.74
M3–O1 × 2	2.220(5)	2.232(7)
M3–O2 × 2	2.210(9)	2.263(10)
M3–O3 × 2	2.112(8)	2.117(11)
Δ(M3O_6_)	5.0×10^–4^	8.1×10^–4^
BVS(M3^2+^)	+2.11	+1.99
Sb–O1 × 2	1.987(5)	1.971(7)
Sb–O2 × 2	1.986(9)	1.960(10)
Sb–O3 × 2	1.981(8)	1.982(10)
Δ(SbO_6_)	1.7×10^–6^	2.1×10^–5^
BVS(Sb^5+^)	+5.35	+5.55
M3–O1–Sb × 2	142.0(4)	141.6(6)
M3–O2–Sb × 2	138.4(3)	135.2(4)
M3–O3–Sb × 2	146.8(3)	144.7(4)

a BVS = ∑_*i*=1_^*N*^ν_*i*_, ν_*i*_ = exp­[(*R*_0_ – *l*_*i*_)/*B*], *N* is the coordination number, *l_i_* is a bond length, *B* = 0.37, *R*_0_(Ce^3+^) = 2.121, *R*_0_(Mn^2+^) = 1.79, *R*_0_(Zn^2+^) = 1.704, *R*_0_(Sb^5+^) = 1.942.

### 3.2 Magnetic Properties

Figure 2 presents the magnetic susceptibility of Ce_2_MnM­(Mn_2_Sb_2_)­O_12_ samples with M = Mn and Zn as a function of temperature at *H* = 100 and 10 kOe under ZFC and FCC conditions. In the 100 Oe ZFC and FCC measurements, both samples display sharp susceptibility increases below *T*_C_ = 52 K (M = Mn) and *T*_C_ = 34 K (M = Zn), suggesting a rapid arrangement of FM-like domains along the direction of the field, where the *T*_C_ values (ferrimagnetic Curie temperatures) were determined from sharp peaks on the ZFC and FCC d*χT*/d*T* versus *T* curves at *H* = 100 and 10 kOe (Figures S3 and S4). Both 100 Oe ZFC and FCC curves of Ce_2_MnMn­(Mn_2_Sb_2_)­O_12_ show a maximum *χ* value at 47 K (*Tχ*_max_), decrease, and then go through a zero point of magnetic susceptibility (*χ* = 0) at *T*_comp_. Below *T*_comp_, the magnetizations (judged from the magnetic susceptibilities) remain negative down to the lowest temperature of ∼2 K, showing the NME or magnetization reversal. The 100 Oe ZFC and FCC curves of Ce_2_MnZn­(Mn_2_Sb_2_)­O_12_ below *Tχ*_max_ (∼20 K, determined from the 100 Oe ZFC curve) follow totally different paths. The ZFC curve shows a maximum at *Tχ*_max_, then decreases and remains negative below *∼*13.6 K. On the other hand, the FCC curve increases steadily as the temperature is decreased and eventually approaches a saturation value at ∼5 K. Notably, no divergence is observed in both samples between the ZFC and FCC curves at 100 Oe down to *Tχ*_max_, while the ZFC and FCC curves nearly overlap under the 10 kOe field. We note that CeO_2_ and Sb_2_O_4+*x*_ impurities are nonmagnetic, and La_3_Mn_2_Sb_3_O_14_-type impurity may be paramagnetic or weakly magnetic. No magnetic transitions were found in La_3_Mn_2_Sb_3_O_14_, and Nd_3_Mn_2_Sb_3_O_14_ showed a magnetic transition near 2 K.^50^ Thus, specific heat anomalies in our samples near 2 K (see below) could originate from La_3_Mn_2_Sb_3_O_14_-type impurity (namely, from a phase with a composition close to Ce_3_Mn_2_Sb_3_O_14_), and similar specific heat anomalies were observed in Nd_2_MnMn­(Mn_4–*x*_Sb_*x*_)­O_12_ samples (with *x* = 1.9 and 2), which also had a similar impurity.^38^ Therefore, impurities should have small effects on the observed magnetic properties.

**Figure 2:**
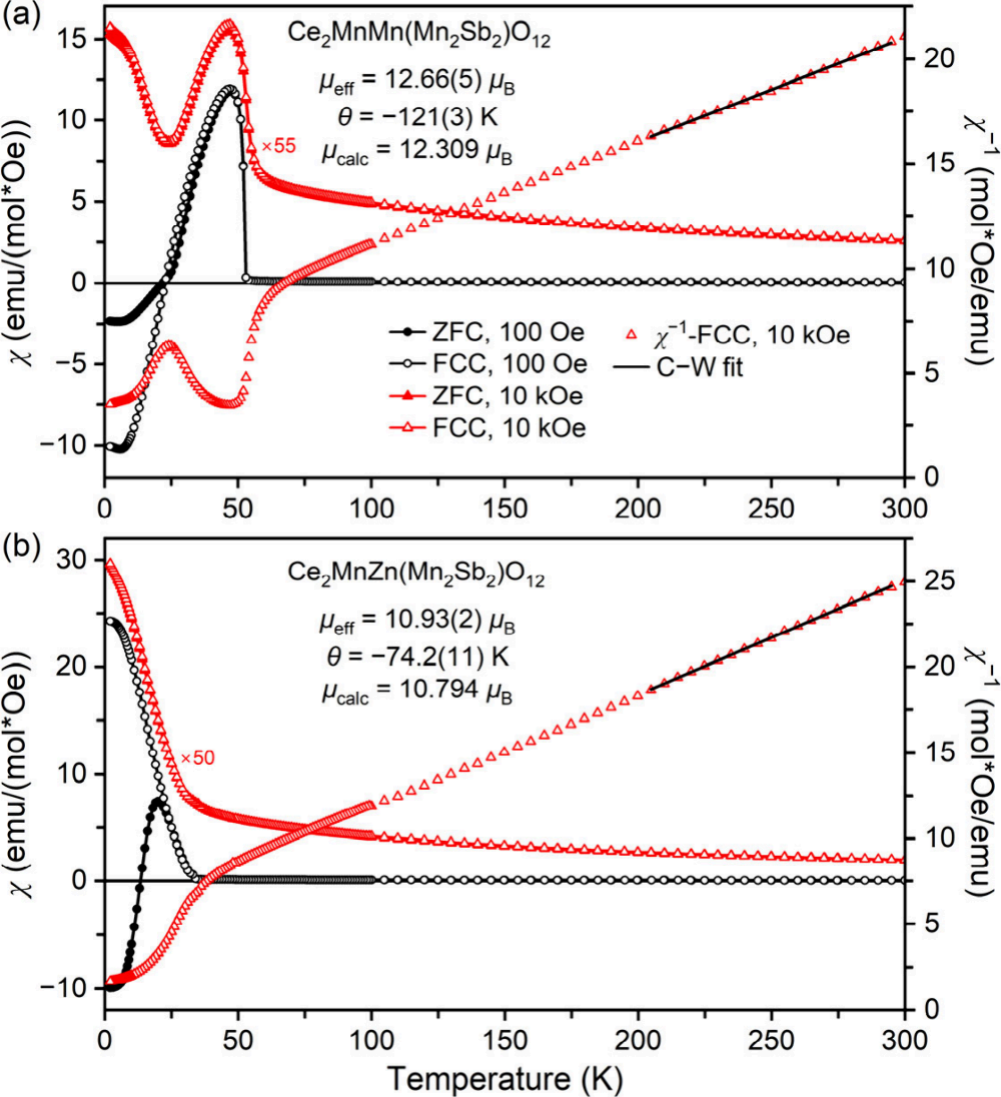
ZFC (filled symbols) and FCC (empty symbols) dc magnetic susceptibility (χ = *M*/*H*) curves of Ce_2_MnM­(Mn_2_Sb_2_)­O_12_ with (a) M = Mn and (b) M = Zn measured at 100 Oe (black, circles) and 10 kOe (red, triangles). The black line gives the Curie–Weiss fit (C-W fit) using the FCC χ^–1^ versus *T* curves at 10 kOe (the right-hand axis).

The inverse magnetic susceptibility was fitted by the Curie–Weiss law at temperatures above 200 K. The Curie–Weiss parameters were obtained from the Curie–Weiss equation fit detailed in Experimental Section and concluded in Table 4. The experimentally determined effective magnetic moments (μ_eff_) closely match the theoretically expected values (μ_calc_).^51^ We note that the inclusion of Ce^3+^ moments (2.4 μ_B_)^51^ gave a better agreement between the calculated and experimental values, thus, giving an indirect support of the oxidation state of Ce. The negative values of the Curie–Weiss temperature (Θ) indicated the dominance of AFM interactions.

**Table 4:** Angles (in deg) Mediating the Main Magnetic Interactions, Ferrimagnetic Curie Temperatures (*T*_C_) and Parameters of Curie–Weiss Fits, and *M* versus *H* Curves at *T* = 5 K for Ce_2_MnM­(Mn_2_Sb_2_)­O_12_ with M = Mn and Zn^a^

	⟨Ce–O–Mn_B_⟩				
M	O1	O2	O3	⟨Mn_A′_–O1–Mn_B_⟩	⟨Mn_A″_–O2–Mn_B_⟩
Mn	83.0(3)	87.1(3)	102.6(3)	103.8(3)	104.7(4)
Zn	81.4(4)	85.5(3)	103.6(4)	103.9(4)	

M	*T*_C_ (K)	μ_eff_ (μ_B_/fu)	μ_calc_ (μ_B_/fu)	θ (K)	*M*_R_ (μ_B_/fu)	*M*_extr_ (μ_B_/fu)	*H*_C_ (kOe)
Mn	52	12.71(5)	12.309	–121(3)	0.221	0.15	1.39
Zn	34	10.98(2)	10.794	–74.2(11)	0.622	0.67	0.48

a Curie–Weiss fits were performed between 200 and 295 K using the FCC *χ* ^–1^ versus *T* data at 10 kOe. *T*_C_ is determined from sharp peaks on the 100 Oe FCC d*χ*/d*T* versus *T* curves. *M*_R_ is the remnant magnetization at *T* = 5 K. *M*_extr_ at *T* = 5 K is obtained by the extrapolation between 40 and 70 kOe to zero field. *H*_C_ is the coercive field at *T* = 5 K.

We emphasize that negative magnetization was also observed on the ZFC curves of Ce_2_MnMn­(Mn_2_Sb_2_)­O_12_ and Ce_2_MnZn­(Mn_2_Sb_2_)­O_12_ measured at 100 Oe (Figure 2). In the majority of cases, negative magnetization on ZFC curves is due to artifacts caused by two main reasons. The first reason is a negative trapped field inside a magnetometer where a negative trapped field produces a negative initial magnetization,^52,53^ which sometimes cannot be switched to a positive value by a small, positive measurement/applied field because of large coercive fields of a material. The second reason is a sample insertion procedure for some models of magnetometers that are kept at low temperatures as the base temperature (for example, at 10 or 100 K). In this case, negative magnetization on ZFC curves was observed even in positive trapped fields because samples were moved through a magnet below magnetic ordering temperatures, and no negative magnetization was observed when samples were moved through a magnet at 300 K.^54^ In our current case, the base temperature of a magnetometer was 300 K; therefore, samples were moved through a magnet at 300 K, which is well above the magnetic ordering temperatures.

To check whether negative magnetization on ZFC curves of Ce_2_MnMn­(Mn_2_Sb_2_)­O_12_ and Ce_2_MnZn­(Mn_2_Sb_2_)­O_12_ was intrinsic, we utilized the following procedure. We cooled samples in applied fields of 10 and −10 Oe (applied after the magnet-reset procedure), which should simulate intentionally large positive and negative trapped fields (PTF and NTF), respectively. At the same time, we measured magnetization down to 2 K (this is equivalent to the FCC measurements at 10 and −10 Oe). As shown in Figure 3, in the paramagnetic states, magnetization of both samples was positive at 10 Oe and negative at −10 Oe, confirming the signs of the “trapped” fields; such FCC curves at 10 and −10 Oe were nearly symmetrical relative to the temperature axis. Then, a measurement field of 100 Oe was applied at 2 K, and the ZFC curves were measured. The absolute values of magnetization did not change much on moving from 10 to 100 Oe (and from −10 to 100 Oe) at 2 K. As a result, magnetization remained negative when the trapped field was positive (10 Oe), and magnetization remained positive when the trapped field was negative (−10 Oe) due to the presence of negative magnetization phenomena in both compounds. Therefore, we can conclude that negative magnetization on ZFC curves in Ce_2_MnMn­(Mn_2_Sb_2_)­O_12_ and Ce_2_MnZn­(Mn_2_Sb_2_)­O_12_ is intrinsic (assuming that fields should approach zero values from the positive direction in all ZFC procedures) and originates from the existence of NME and finite positive trapped fields inside a magnetometer. It is interesting that magnetization changed sign two times (at 23 and 30 K) in the case of Ce_2_MnMn­(Mn_2_Sb_2_)­O_12_ when the “trapped” field was negative (−10 Oe) because the ZFC *M* versus *T* curve (at 100 Oe) followed the FCC *M* versus *T* curve (at −10 Oe) up to a certain temperature (about 25 K); the point where these curves are separated could be related to temperature dependence of coercive fields. For example, these curves start to separate above about 7 K in Ce_2_MnZn­(Mn_2_Sb_2_)­O_12_. Therefore, ZFC curves of the M = Zn sample (obtained under negative trapped fields) do not show negative values, and the “compensation” temperature (about 13 K) on ZFC curves of the M = Zn sample (obtained under positive trapped fields) does not match with the compensation temperature of the FCC curves.

**Figure 3:**
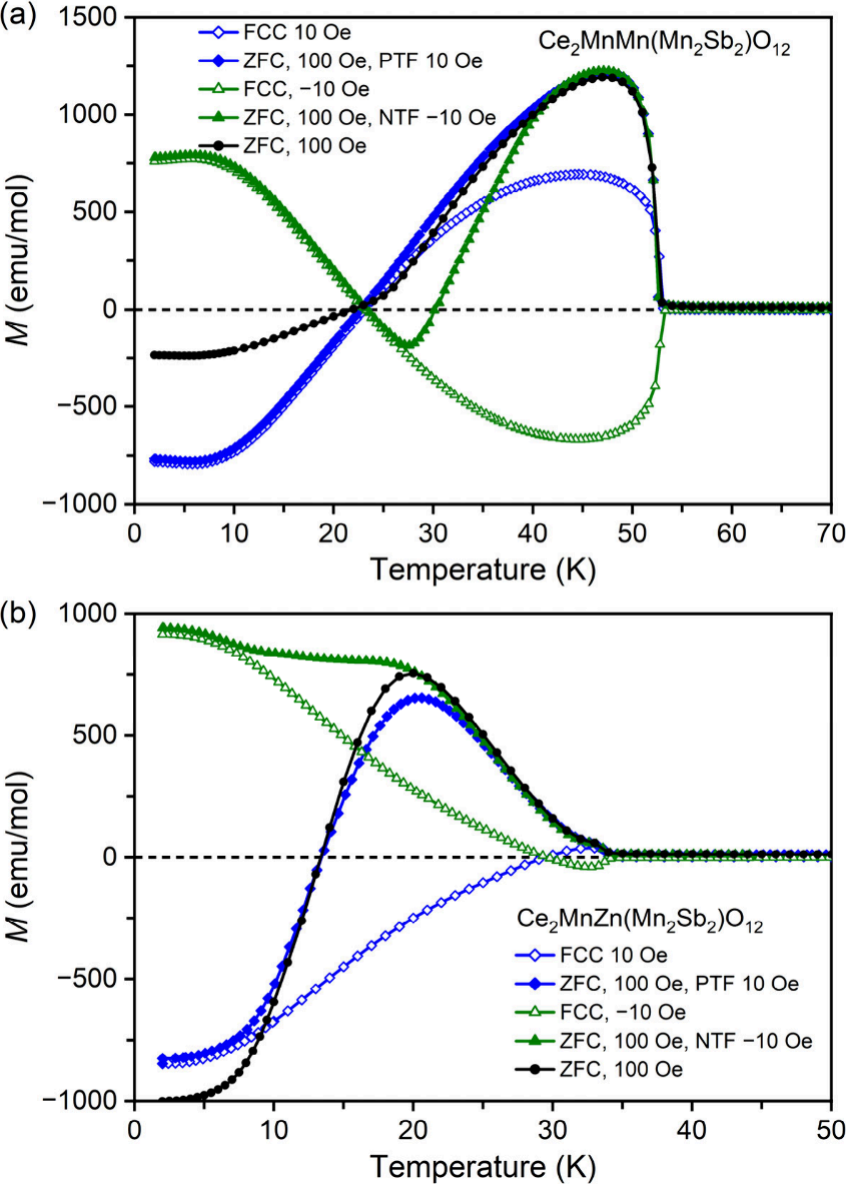
Magnetization (*M* versus *T*) curves of Ce_2_MnM­(Mn_2_Sb_2_)­O_12_ with (a) M = Mn and (b) M = Zn measured at different conditions and fields. PTF indicates a positive trapped field, and NTF represents a negative trapped field.

To further explore the NME in Ce_2_MnMn­(Mn_2_Sb_2_)­O_12_ and Ce_2_MnZn­(Mn_2_Sb_2_)­O_12_, FCC magnetic susceptibility measurements were performed under various applied magnetic fields. As shown in Figure 4a, the magnetic susceptibility of Ce_2_MnMn­(Mn_2_Sb_2_)­O_12_ exhibits a consistent trend across different fields: a rapid increase below *T*_C_, reaching a peak at *Tχ*_max_ (47 K), followed by a decrease. NME is observed at low temperatures when *H* ≤ 600 Oe, with *T*_comp_ shifting to lower values as the field increases (inset of Figure 4a) and a reduction in the NME component. For *H* ≥ 800 Oe, only positive magnetization is observed, and the susceptibility shows a slight increase at ∼23 K. Furthermore, the maximum values of the FCC curves above *T*_comp_ increase as the field decreases. As will be further discussed below, the NME in Ce_2_MnMn­(Mn_2_Sb_2_)­O_12_ likely arises from FM ordering of Mn^2+^ at the A′ and A″ sites and an AFM arrangement of Mn^2+^ at the B site.^34,36^ These competing magnetic interactions give rise to complex temperature-dependent magnetization behavior. In contrast, Ce_2_MnZn­(Mn_2_Sb_2_)­O_12_ shows NME only at a significantly lower field range (*H* ≤ 20 Oe), with *T*_comp_ decreasing from 29.6 K at *H* = 10 Oe to 28.5 K at *H* = 20 Oe) (Figure 4b). Below *T*_C_, the magnetic susceptibility rapidly peaks at *T*χ_max_ = 32.6 K, followed by either a continuous increase (*H* ≥ 40 Oe) or a decrease (*H* ≤ 20 Oe). The suppressed NME in Ce_2_MnZn­(Mn_2_Sb_2_)­O_12_ can be attributed to the presence of nonmagnetic Zn^2+^ ions, which do not participate in magnetic exchange interactions.

**Figure 4:**
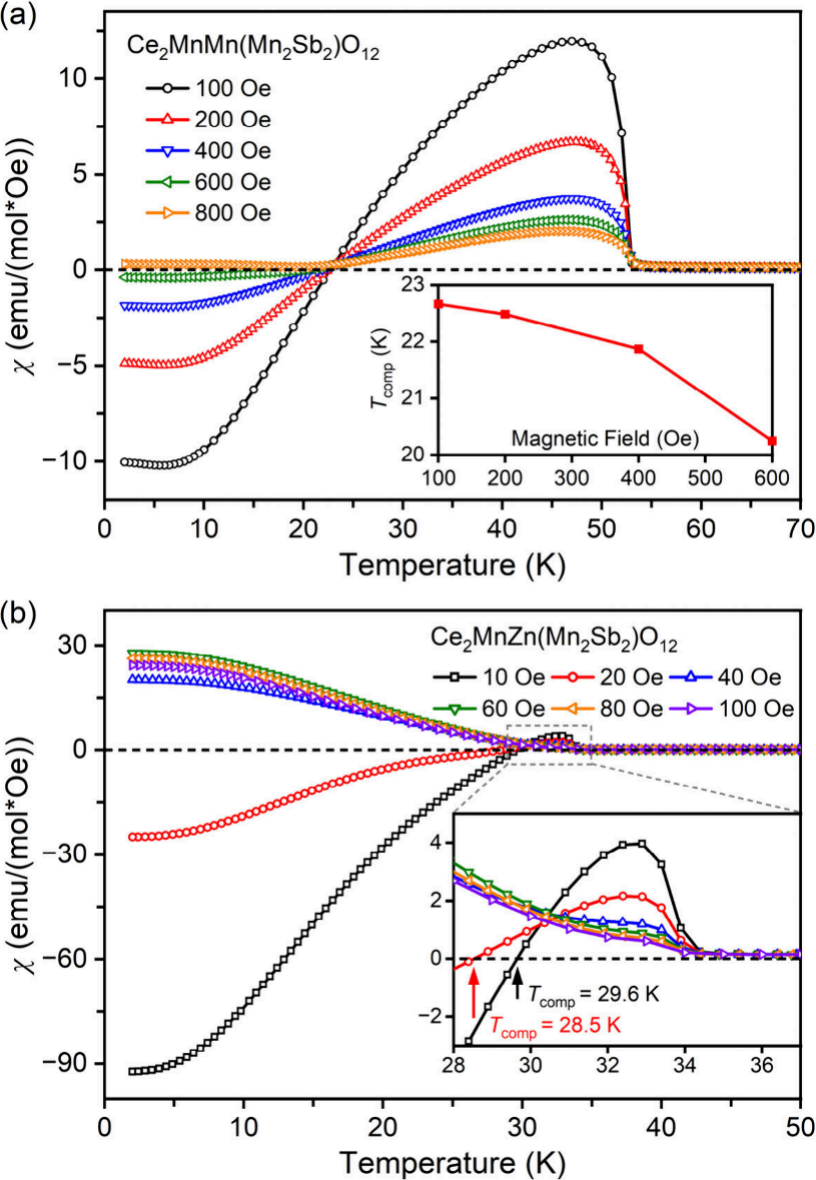
FCC dc magnetic susceptibility (χ = *M*/*H*) curves of Ce_2_MnM­(Mn_2_Sb_2_)­O_12_ with (a) M = Mn and (b) M = Zn measured at different fields. The inset in (a) shows the plot of the compensation temperature (*T*_comp_) as a function of the applied magnetic field. The inset in (b) shows a zoomed-in region near the Curie temperature (*T*_C_).

Isothermal magnetization curves (*M* versus *H*) at different temperatures are presented in Figure 5, with the corresponding parameters being summarized in Table 4. The presence of hysteresis substantiates the presence of FM components in magnetic ordering. At 5 K, both samples exhibit an incomplete wasp-waisted shape with low remnant magnetization (*M*_R_) and coercive fields (*H*_C_), attributed to the coexistence of competing FM and AFM interactions.^55^ The unsaturated behavior of both samples under high magnetic fields indicates that AFM coupling is the dominant factor influencing their magnetic properties. The extrapolated magnetization (*M*_extr_) determined via linear extrapolation is quite small, reflecting the presence of strong magnetic competition and complex lattice coupling. As the temperature increases, the hysteresis loop narrows and *H*_C_ decreases (see insets of Figure 5 and Figures S5 and S6). Notably, a slight increase in *M*_*R*_ is observed around 40 K of Ce_2_MnMn­(Mn_2_Sb_2_)­O_12_, which corresponds to an anomaly in the 10 kOe ZFC and FCC curves (Figure 2a).

**Figure 5:**
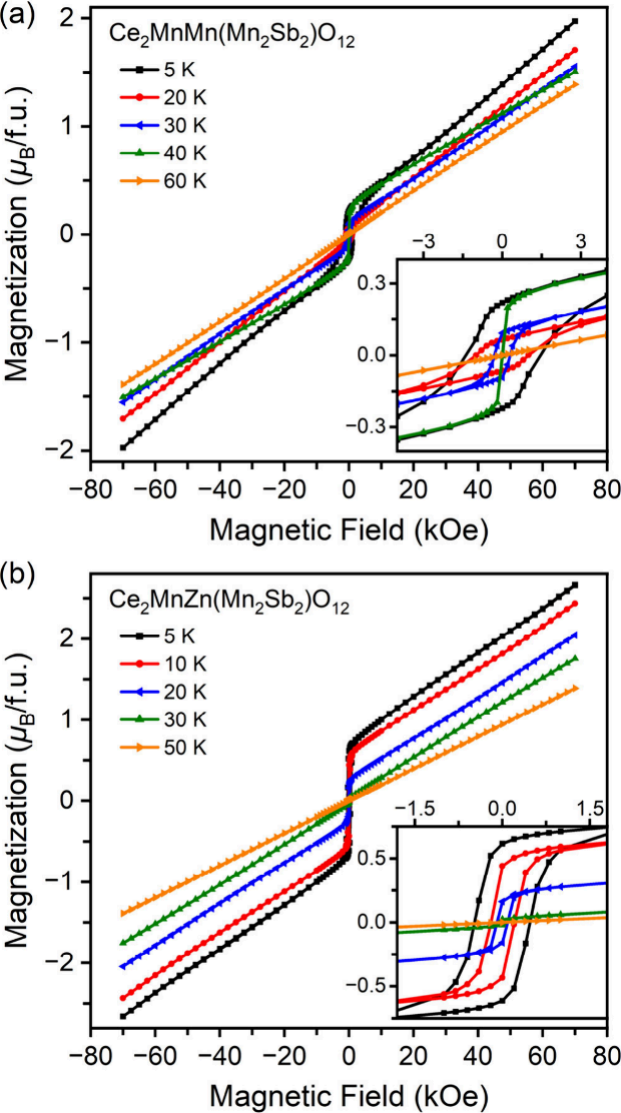
*M* versus *H* curves of Ce_2_MnM­(Mn_2_Sb_2_)­O_12_ with (a) M = Mn and (b) M = Zn at different temperatures. Each inset shows a zoomed-in region near zero field.

The specific heat (*C*_p_) measurements at *H* = 0 and 90 kOe for Ce_2_MnMn­(Mn_2_Sb_2_)­O_12_ and Ce_2_MnZn­(Mn_2_Sb_2_)­O_12_ are presented in Figure 6a. Pronounced anomalies are observed at their respective *T*_C_ values, confirming the presence of long-range magnetic ordering. These anomalies reflect an increase in magnetic entropy as the system transitions from an ordered state to a disordered state. Interestingly, while the magnetic transition in Ce_2_MnMn­(Mn_2_Sb_2_)­O_12_ is suppressed under a field of 90 kOe, the transition in Ce_2_MnZn­(Mn_2_Sb_2_)­O_12_ remains relatively unchanged. This difference may be due to the substitution of Mn by Zn, which reduces the number of magnetic ions and potentially weakens the AFM coupling, leading to a reduced response of the magnetic order to external fields in Ce_2_MnZn­(Mn_2_Sb_2_)­O_12_. There is evidence of the appearance of the second magnetic transition near 40 K in Ce_2_MnMn­(Mn_2_Sb_2_)­O_12_ at 90 kOe (Figure 6a). In magnetic insulators, the *C*_p_ capacity is predominantly governed by lattice vibrations at higher temperatures and magnetic excitations at lower temperatures. To isolate the magnetic contribution from the total *C*_p_, the lattice contribution (*C*_lattice_) was estimated using the experimentally measured *C*_p_ of Nd_2_ZnZn­(Zn_2_Sb_2_)­O_12_, a compound with the same structure and paramagnetic behavior down to 2 K (unpublished data) (Figures S7 and S8). This paramagnetic material features nonmagnetic cations occupying the A′, A″, and B sites. The magnetic part of the heat capacity is calculated by subtracting the lattice part, *C*_mag_ = *C*_p_ – *C*_lattice_ (the left axis of Figure 6b). The magnetic entropy (*S*_mag_) was subsequently determined by integrating *C*_mag_/*T* (the right axis of Figure 6b). The expected magnetic entropy for the Mn^2+^ moment is *S*_mag_ = *R*ln­(2*J* + 1) = *R*ln6 ≈ 14.89 J K^–1^ mol^–1^, where *R* = 8.314 J K^–1^ mol^–1^ is the universal gas constant. The *S*_mag_ for both samples gradually increases at low temperatures, nearing saturation around 100 K. At *T* = 100 K, *S*_mag_ for Ce_2_MnMn­(Mn_2_Sb_2_)­O_12_ is 51.2 J K^–1^ mol^–1^, while that for Ce_2_MnZn­(Mn_2_Sb_2_)­O_12_ is 40.5 J K^–1^ mol^–1^. These values are slightly lower than the expected Boltzmann entropy estimated from the Mn content (4*R*ln6 and 3*R*ln6, Figure 6) due to difficulties in the precise estimation of the lattice contribution. The difference between the two is consistent with the reduction in *S*_mag_ due to the substitution of Mn by Zn at the A″ site, which reduces the number of magnetic moments contributing to the total entropy.

**Figure 6:**
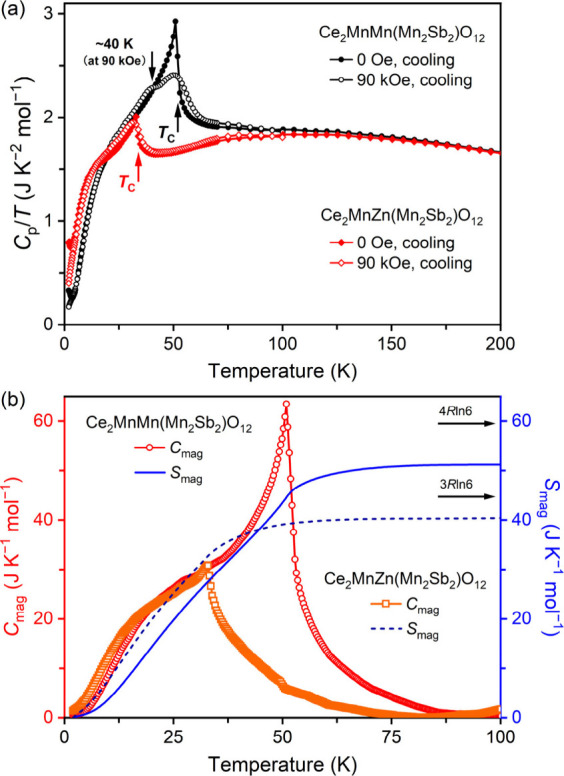
(a) Specific heat data of Ce_2_MnM­(Mn_2_Sb_2_)­O_12_ with M = Mn and Zn plotted as *C*_p_/*T* versus *T*. Measurements were performed on cooling at *H* = 0 and 90 kOe. (b) Temperature dependences of the magnetic contribution to the heat capacity (*C*_mag_) and the magnetic entropy (*S*_mag_) of Ce_2_MnM­(Mn_2_Sb_2_)­O_12_ with M = Mn and Zn at *H* = 0 Oe.

### 3.3 Discussion

The NME observed in Ce_2_MnM­(Mn_2_Sb_2_)­O_12_ (M = Mn, Zn) represents a remarkable phenomenon arising from the intricate interplay between magnetic sublattices and the crystal structure. Such a system belongs to one of the five known NME mechanisms,^3^ specifically those involving antiparallel ordering between two (or more) FM sublattices associated with distinct crystallographic sites. This discussion first addresses the mechanism of NME in Ce_2_MnMn­(Mn_2_Sb_2_)­O_12_ and then illustrates how substituting Mn^2+^ with nonmagnetic Zn^2+^ at the A″ site affects NME in Ce_2_MnZn­(Mn_2_Sb_2_)­O_12_. The type and relative strength of magnetic interactions in both materials are critically determined by the geometry of the M–O–M superexchange pathways, with the corresponding angles summarized in Table 4. In Ce_2_MnMn­(Mn_2_Sb_2_)­O_12_, the rock-salt ordering of B site Mn^2+^ and Sb^5+^ cations necessitates the involvement of super-superexchange Mn_B_–O–Sb–O–Mn_B_ pathways; furthermore, the octahedral geometry of B site Mn^2+^ minimizes interactions with neighboring B sites. The square-planar coordination of the A′ site Mn^2+^ and the tetrahedral coordination of the A″ site Mn^2+^ prevent direct magnetic interactions between equivalent A′ and A″ sites. Consequently, the magnetic behavior of Ce_2_MnMn­(Mn_2_Sb_2_)­O_12_ is dominated by two key AFM superexchange pathways: Mn_A′_–O–Mn_B_ and Mn_A″_–O–Mn_B_. The respective superexchange bond angles support moderate AFM coupling, consistent with the Goodenough −Kanamori–Anderson (GKA) rules.^56^ A′ and A″ site Mn^2+^ moments align parallel to each other but antiparallel to B site Mn^2+^ moments, creating a FIM lattice.^36,55^ The AFM coupling mechanism has been experimentally confirmed in related compounds through neutron powder diffraction (NPD) studies, including R_2_MnMn­(Mn_2_Sb_2_)­O_12_ with R = La, Pr, and Nd.^36,55^

The schematic illustration of the spin configurations in Ce_2_MnMn­(Mn_2_Sb_2_)­O_12_ is presented in Figure 7, and the temperature-dependent magnetic behavior exhibits four distinct regimes. (i) *T* > *T*_C_: Above *T*_C_, thermal agitation dominates, leading to paramagnetic behavior without long-range magnetic ordering. (ii) *T*χ_max_ < *T* < *T*_C_: As the system cools below *T*_C_, magnetic ordering develops. The A′ and A″ site Mn^2+^ moments (*M*_MnA′,A″_) grow rapidly due to strong exchange interactions, while B site Mn^2+^ moments (*M*_MnB_) increase more slowly due to dilution of the whole B sublattice. The sublattice moments align along the easy axis, which is dictated by magnetic anisotropy. Net magnetization (*M*_net_) remains positive but small due to the imbalance between sublattices. At *T*χ_max_, magnetic susceptibility peaks, reflecting the system’s heightened responsiveness to external fields. The near invariance of *T*χ_max_ across different fields (Figure 4a) suggests that local AFM coupling and thermal agitation, rather than external fields, govern this characteristic temperature. (iii) *T*_comp_ < *T* < *T*χ_max_: As the temperature decreases further, *M*_MnB_ grows significantly faster than *M*_MnA′,A″_. The 4-fold coordination of both A′ and A″ sites imposes strong local anisotropies that hinder rapid magnetic reorientation, enabling *M*_MnB_ to dominate. This imbalance causes *M*_net_ to decrease, reaching zero at *T*_comp_ as opposing sublattice moments cancel out (*M*_MnA′,A″_ = *M*_MnB_). (iv) *T* < *T*_comp_: Below *T*_comp_, *M*_MnB_ dominates, leading to negative *M*_net_. Strong AFM coupling between the B and A′/A″ sites suppresses further alignment with external fields. This transition aligns with Néel theory,^57^ underscoring the roles of sublattice interactions and anisotropy in determining magnetic behavior.

**Figure 7:**
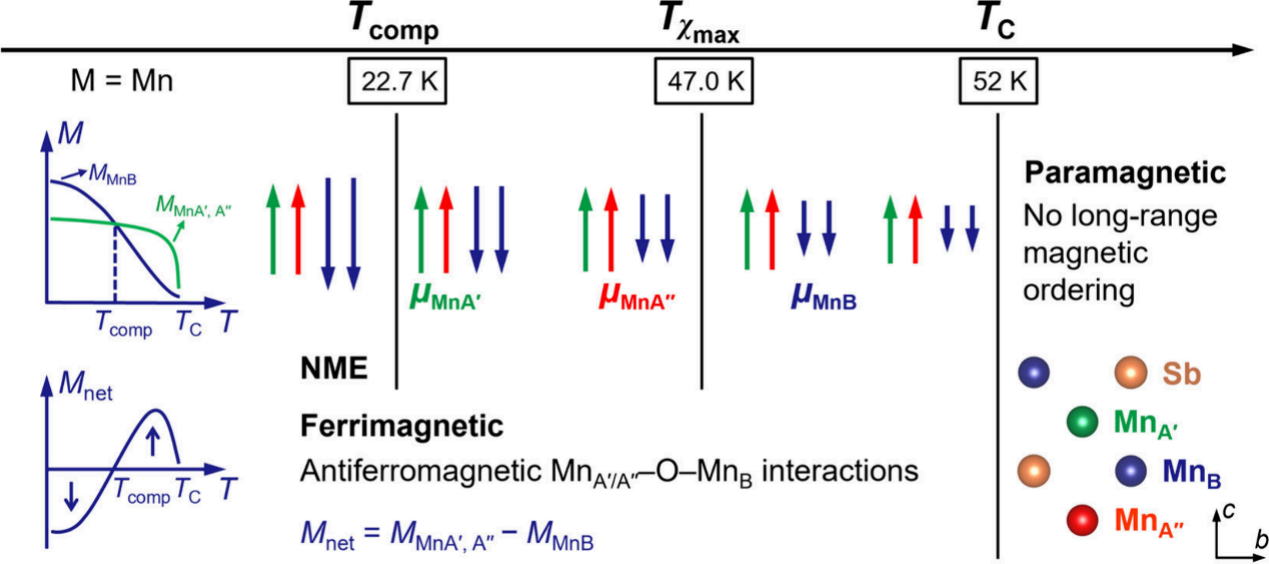
Schematic illustration of the spin configurations in Ce_2_MnMn­(Mn_2_Sb_2_)­O_12_. NME denotes the negative magnetization effect.

The influence of external magnetic fields on the NME is evident across these regimes. At low measurement magnetic fields (below coercive fields), one sublattice overcomes another sublattice in each domain, and the magnetic field cannot switch magnetization in domains, resulting in a smooth *M* versus *T* (or *χ* versus *T*) curves across *T*_comp_. On the other hand, an apparent broad maximum appears in larger magnetic fields (Figure 2a). For example, the observed maximum on the 10 kOe ZFC and FCC curves of Ce_2_MnMn­(Mn_2_Sb_2_)­O_12_ is a typical feature of FIM materials with NME.^13,58^ A low-temperature minimum is also observed very close to *T*_comp_ (when the measurement field is not too high). The origin of this behavior lies again in the NME, when the magnetic field is high enough (above coercive fields) to switch magnetization in domains to the opposite direction at *T*_comp_ because states with negative magnetization relative to the direction of a magnetic field are energetically unfavorable. One sublattice continues to overcome another sublattice or the absolute values of magnetization continue to increase below *T*_comp_ at any magnetic field.

Similar behavior of χ versus *T* curves was observed in MnLaMnSbO_6_,^36,55^ when measured at 1 kOe, with a broad maximum below *T*_C_ = 48 K and a sharp upturn below 9 K. This is a typical feature of FIM materials with NME.^13,58^ Therefore, MnLaMnSbO_6_ could also exhibit NME below 9 K at lower magnetic fields, and the ordered moments of Mn^2+^ at A′, A″, and B sites could be different in contrast to the assumptions of refs 36 and 55 . Sm^3+^ cations usually show no or weak ordered magnetic moments in perovskite oxides,^59^ and MnSmMnSbO_6_ also demonstrated a broad maximum on its χ versus *T* curves (at 1 kOe).^36^ On the other hand, MnRMnSbO_6_ compounds from the same series with detectable R^3+^ ordered moments (R = Pr and Nd)^36,55^ showed no (direct or indirect) signs of NME. Therefore, we can assume that Ce^3+^ cations in Ce_2_MnMn­(Mn_2_Sb_2_)­O_12_ should have no or very weak ordered magnetic moments in order to show NME, as this effect appears to be a competition of ordered moments of Mn at the A′, A″, and B sites. We note that NME was observed in Sm_2_MnMn­(Mn_2_Ti_2_)­O_12_.^59^ However, another sample^34^ with the same composition did not show NME, suggesting that small variations in cation distributions could play a major role in the appearance of NME.

Substituting Mn^2+^ at the A″ site with nonmagnetic Zn^2+^ in Ce_2_MnZn­(Mn_2_Sb_2_)­O_12_ significantly affects its magnetic properties. Zn substitution eliminates the A″–O–B AFM pathway, reducing the overall AFM coupling. This weakens sublattice competition, leading to higher remnant magnetization and diminished NME, as observed in Ce_2_MnZn­(Mn_2_Sb_2_)­O_12_. Moreover, the *T*_C_ and *T*_comp_ temperatures are closer in Ce_2_MnZn­(Mn_2_Sb_2_)­O_12_ than in Ce_2_MnMn­(Mn_2_Sb_2_)­O_12_. This proximity suggests that just below *T*_C_, a larger moment is induced on the A′ site than on the B sites. However, as there are no magnetic cations at the A″ site in Ce_2_MnZn­(Mn_2_Sb_2_)­O_12_, the *M*_MnB_ (from two Mn^2+^ cations) quickly overtakes the *M*_MnA′_ (from one Mn^2+^ cation), resulting in *T*_comp_ being very close to *T*_C_. On the other hand, in Ce_2_MnMn­(Mn_2_Sb_2_)­O_12_, the presence of magnetic Mn^2+^ cations at both the A′ and A″ sites leads to a more competitive interaction with the two Mn^2+^ cations at the B sites within the FIM structure. This competition allows *M*_MnB_ to surpass *M*_MnA′,A″_ at a much lower temperature, resulting in a more pronounced separation between *T*_C_ and *T*_comp_. These findings highlight the critical role of cation composition and site occupancy in dictating the magnetic behavior and NME of these complex perovskite-like oxides. We note that broad maxima were also observed in Ce_2_MnZn­(Mn_2_Sb_2_)­O_12_ between *T*_C_ and *T*_comp_ on χ versus *T* curves when measured at magnetic fields above 40 Oe (the inset of Figure 4b) in agreement with the general tendency discussed in the above paragraph. However, because *T*_C_ and *T*_comp_ are very close to each other, the maxima are very small.

## 4 Conclusions

In conclusion, this study presents the observation of the pronounced negative magnetization effect (NME) in A-site columnar-ordered quadruple perovskites, specifically Ce_2_MnM­(Mn_2_Sb_2_)­O_12_ (M = Mn and Zn). These compounds crystallize in the *P*4_2_/*n* (No. 86) space group, demonstrating the complete rock-salt ordering of Mn and Sb at the B sites. The bond-valence sum analysis and charge balance confirm that cerium exists in the +3 oxidation state. The compounds exhibit distinct magnetic transitions at *T*_C_ = 52 K for M = Mn and *T*_C_ = 34 K for M = Zn. Field-cooled measurements under small magnetic fields reveal pronounced NME, which is further corroborated by zero-field-cooled curves under similar conditions. The observed NME is likely a consequence of the ferrimagnetic structures inherent to these materials. These findings underscore the critical influence of complex magnetic interactions and anisotropy coupling in governing their unique magnetic properties.

## Supplementary Material







## Notes

Magnified figures for Rietveld refinements, laboratory XRPD patterns of the samples prepared at different conditions, differential magnetic susceptibility curves, coercive fields as a function of temperature, and specific heat and magnetic entropy change as a function of temperature (PDF)
Crystallographic parameters of main and impurity phases (PDF)
Crystallographic parameters of main and impurity phases (PDF)
