# Supplementary material for: Negative Magnetization Phenomena in A‑Site Columnar-Ordered Quadruple Perovskites Ce2MnM(Mn2Sb2)O12 with M = Mn and Zn
Source: Inorg Chem. 2025 May 16;64(21):10467–77. doi: 10.1021/acs.inorgchem.5c00653 (PMC12135034; doi:10.1021/acs.inorgchem.5c00653)
Supplement: Supplementary file 1 [file ic5c00653_si_001.pdf]

# Supporting Information

## Negative Magnetization Phenomena in A-site Columnar-Ordered Quadruple Perovskites $Ce_2MnM(Mn_2Sb_2)O_{12}$ with M = Mn and Zn

Xuan Liang [a,b], Kazunari Yamaura [a,b], Alexei A. Belik [a,*]

[a] *Research Center for Materials Nanoarchitectonics (MANA), National Institute for Materials Science (NIMS), Namiki 1-1, Tsukuba, Ibaraki 305-0044, Japan*

[b] *Graduate School of Chemical Sciences and Engineering, Hokkaido University, North 10 West 8, Kita-ku, Sapporo, Hokkaido 060-0810, Japan*

* Corresponding author.

E-mail address: Alexei.Belik@nims.go.jp (A.A. Belik)

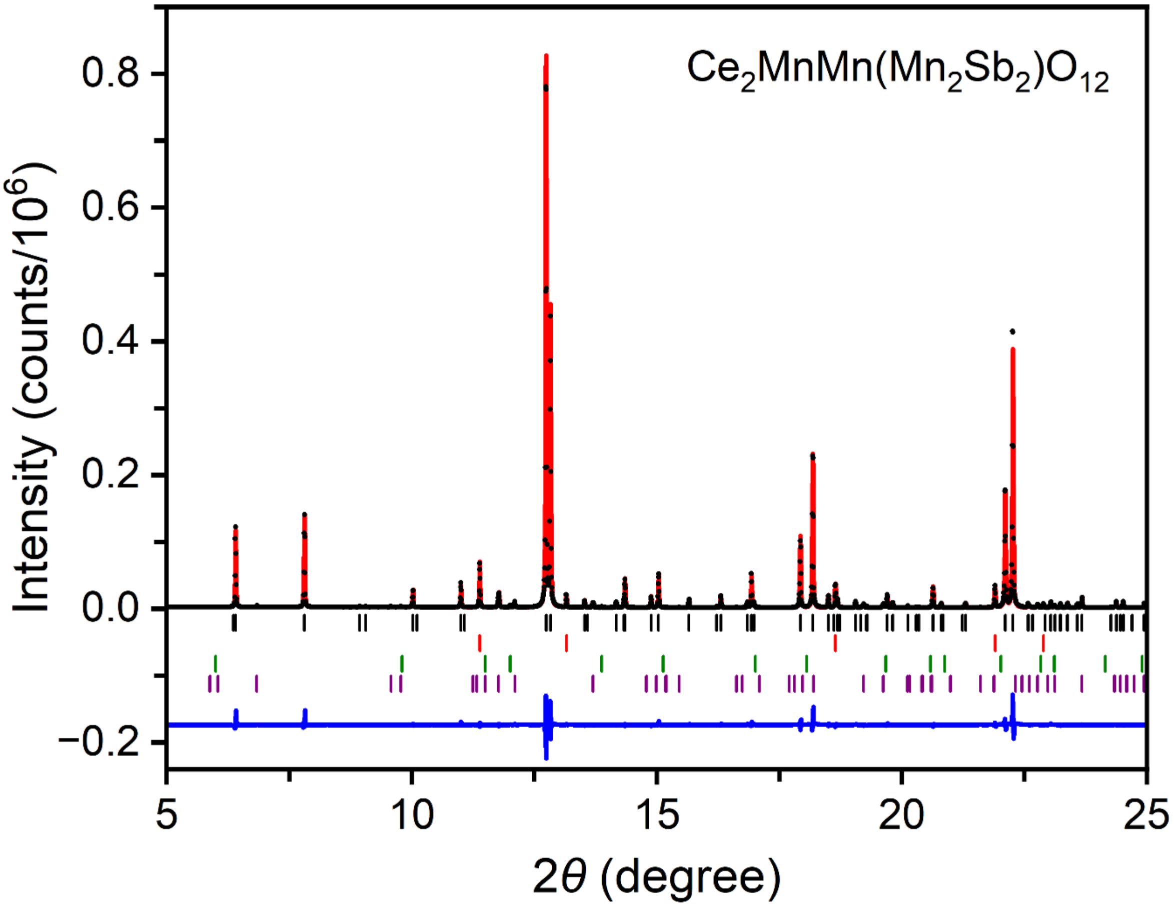


**Figure S1a.** Zoomed-in experimental (black circles), calculated (red line), and difference (blue line at the bottom) synchrotron X-ray powder diffraction patterns of $Ce_2MnMn(Mn_2Sb_2)O_{12}$ in the 5°–25° 2$\theta$ range. The tick marks show possible Bragg reflection positions of the main perovskite phase (the first black row), $CeO_2$ impurity (the second red row), cubic pyrochlore impurity (the third green row), and $La_3Mn_2Sb_3O_{14}$-related pyrochlore impurity (the fourth purple row).

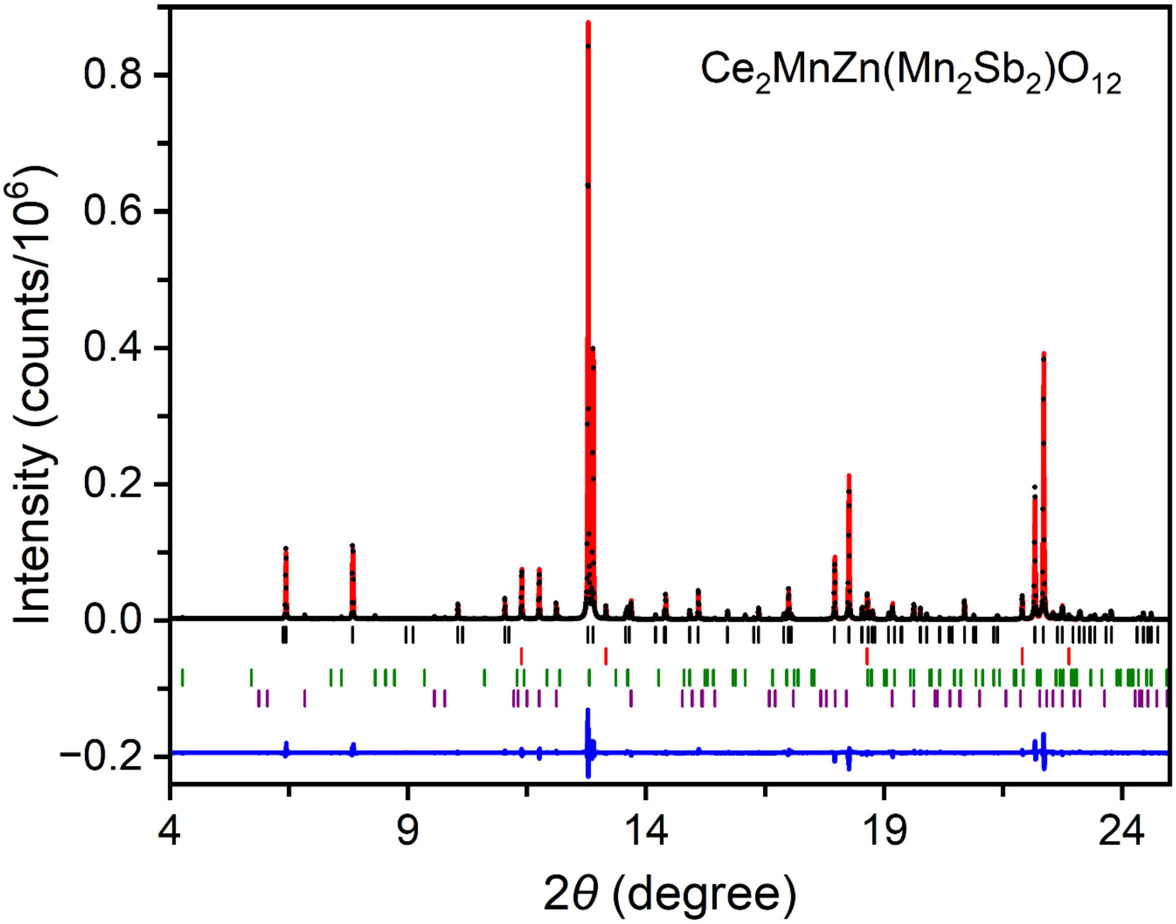


**Figure S1b.** Zoomed-in experimental (black circles), calculated (red line), and difference (blue line at the bottom) synchrotron X-ray powder diffraction patterns of $Ce_2MnZn(Mn_2Sb_2)O_{12}$ in the 4°–25° 2$\theta$ range. The tick marks show possible Bragg reflection positions of the main perovskite phase (the first black row), $CeO_2$ impurity (the second red row), $Na_5Co_{15.5}Te_6O_{36}$-type impurity (the third green row), and $La_3Mn_2Sb_3O_{14}$-related pyrochlore impurity (the fourth purple row).

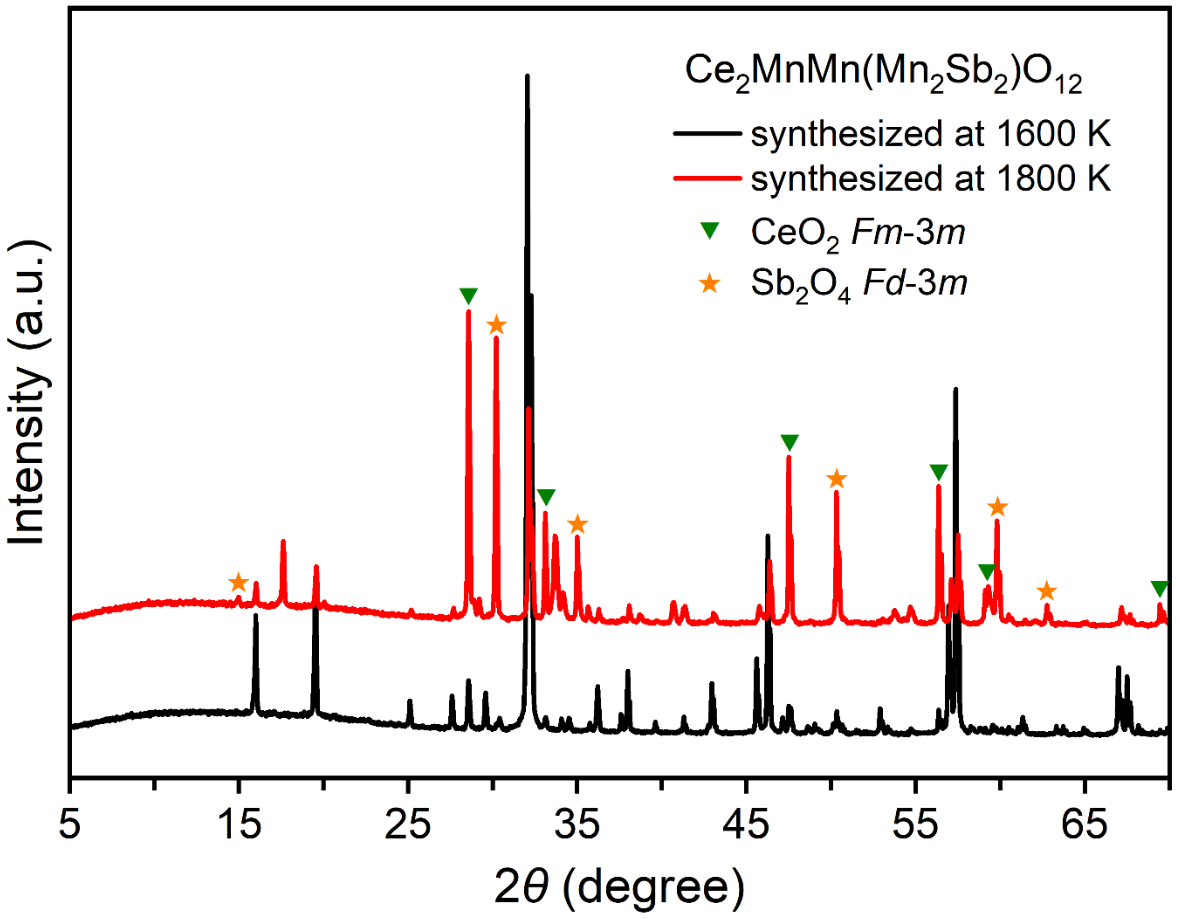


**Figure S2a.** Comparison of laboratory X-ray powder diffraction (XRPD) patterns of $Ce_2MnMn(Mn_2Sb_2)O_{12}$ prepared under 1600 K and 1800 K (at 6 GPa for 2 h). The green triangles show characteristic peaks of $CeO_2$ (space group *Fm*-3*m*) and the orange stars show characteristic peaks of $Sb_2O_4$ (space group *Fd*-3*m*).

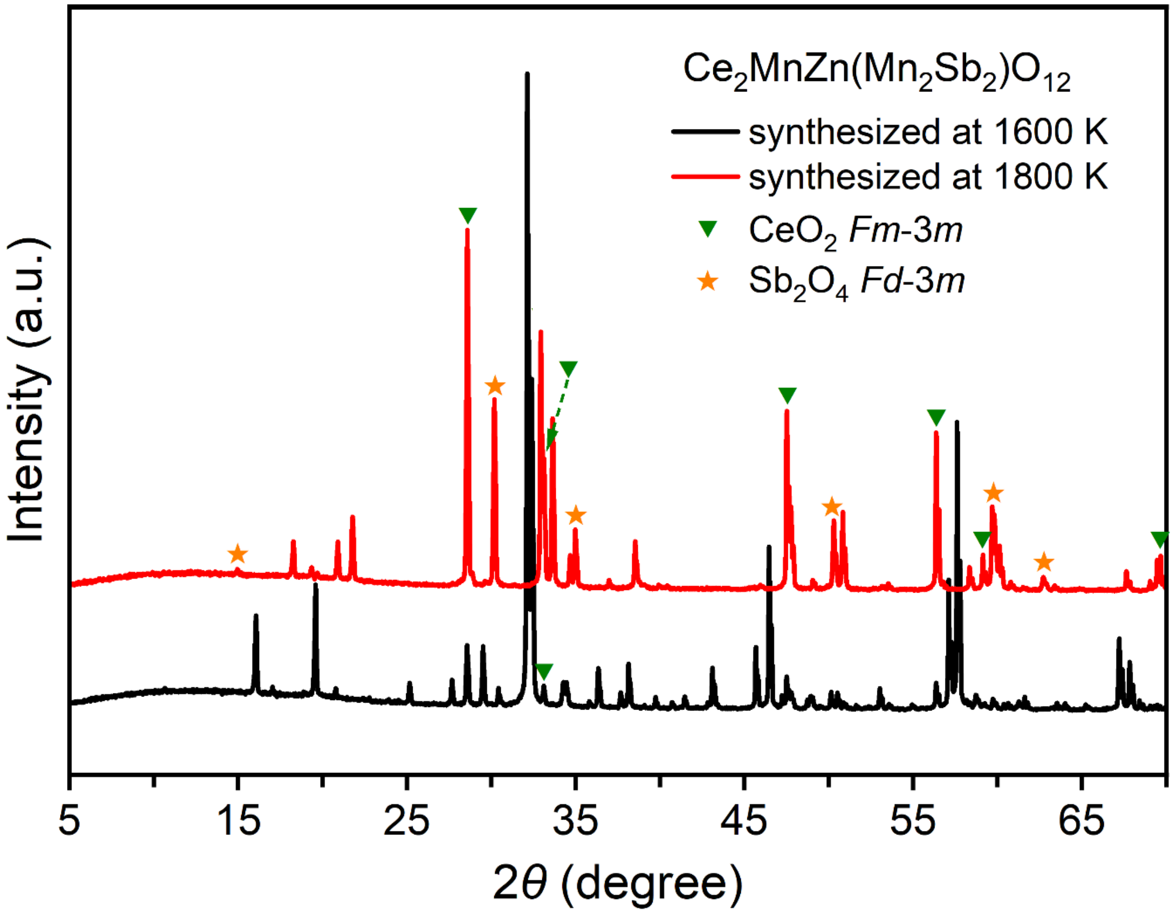


**Figure S2b.** Comparison of laboratory X-ray powder diffraction (XRPD) patterns of $Ce_2MnZn(Mn_2Sb_2)O_{12}$ prepared under 1600 K and 1800 K (at 6 GPa for 2 h). The green triangles show characteristic peaks of $CeO_2$ (space group *Fm*-3*m*) and the orange stars show characteristic peaks of $Sb_2O_4$ (space group *Fd*-3*m*).

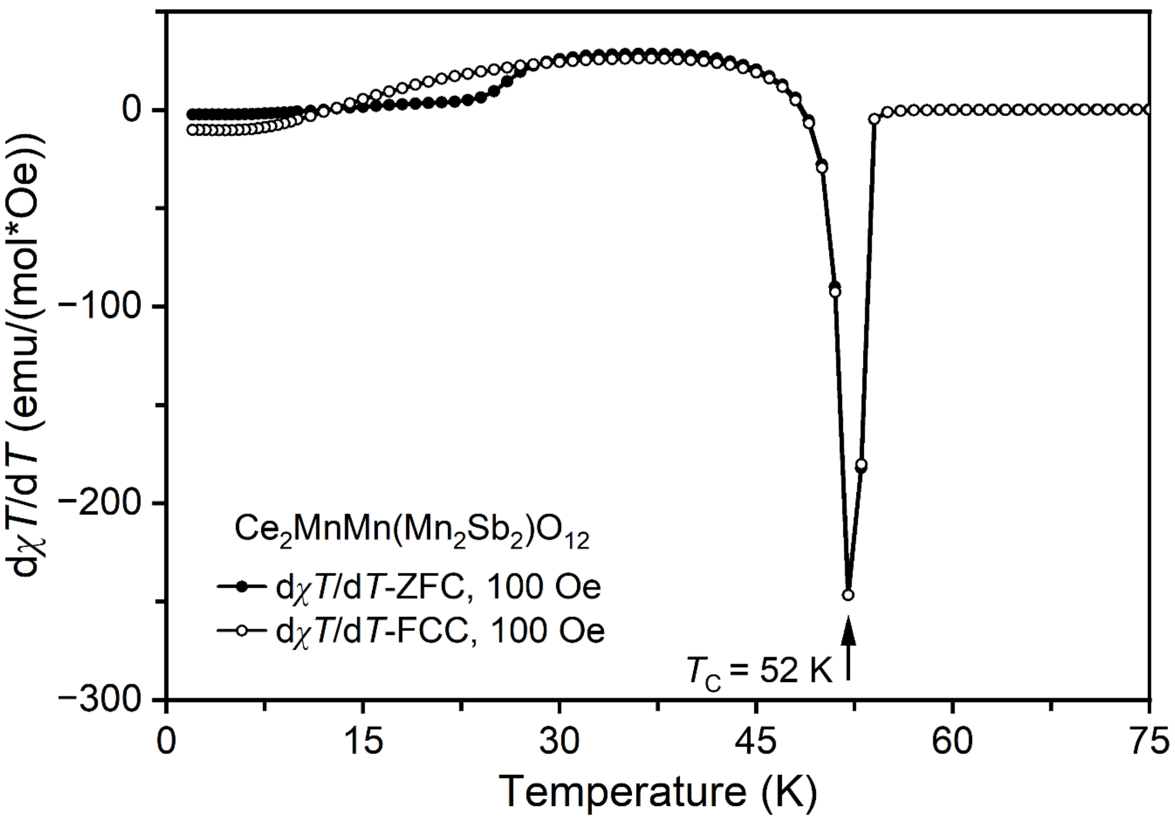


**Figure S3a.** ZFC and FCC d$\chi T$/d$T$ versus $T$ curves of $Ce_2MnMn(Mn_2Sb_2)O_{12}$ at $H$ = 100 Oe.

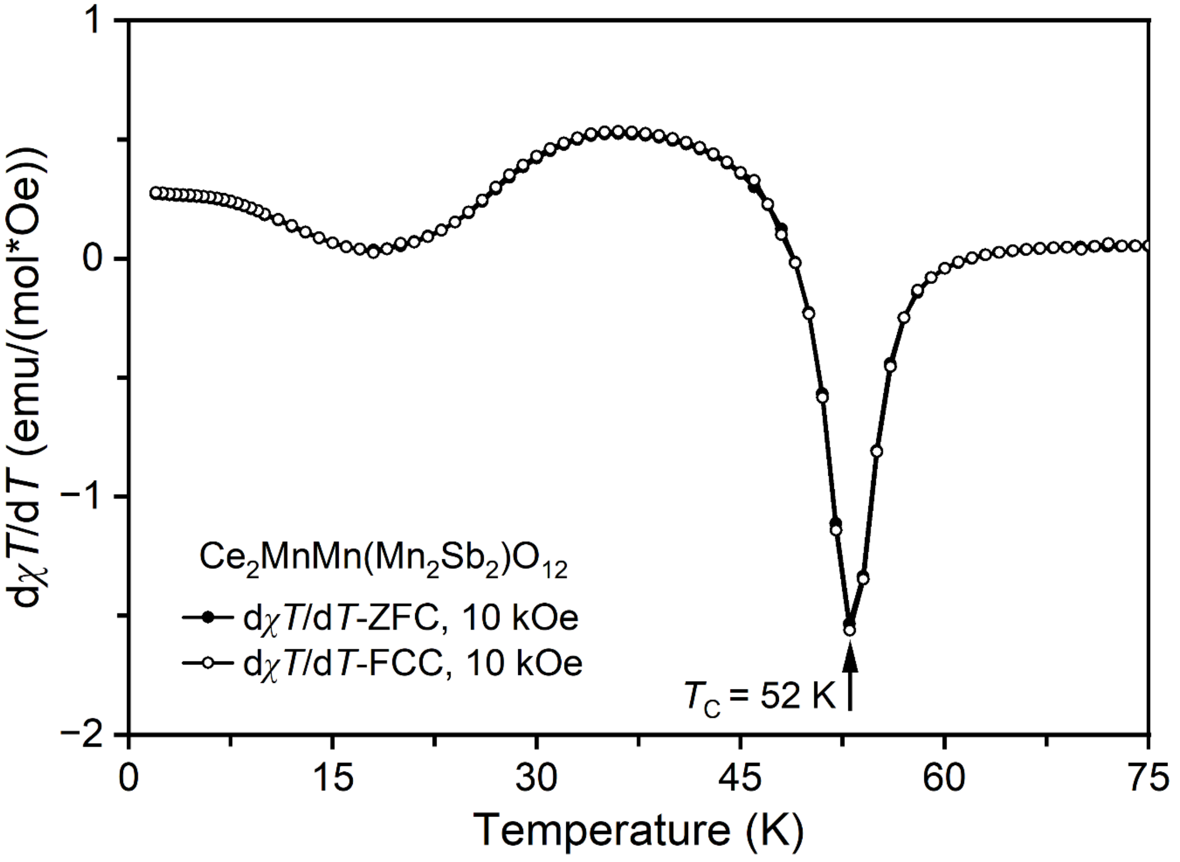


**Figure S3b.** ZFC and FCC d$\chi T$/d$T$ versus $T$ curves of $Ce_2MnMn(Mn_2Sb_2)O_{12}$ at $H$ = 10 kOe.

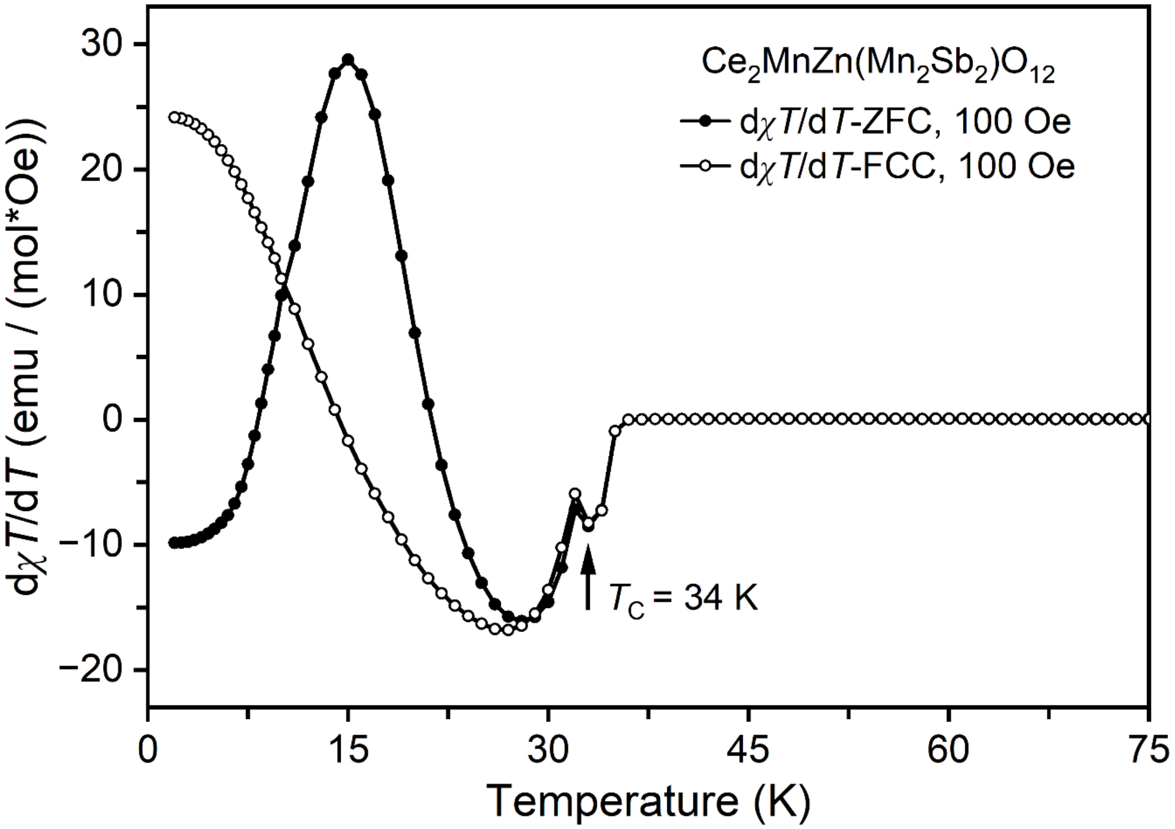


**Figure S4a.** ZFC and FCC d$\chi T$/d$T$ versus $T$ curves of $Ce_2MnZn(Mn_2Sb_2)O_{12}$ at $H$ = 100 Oe.

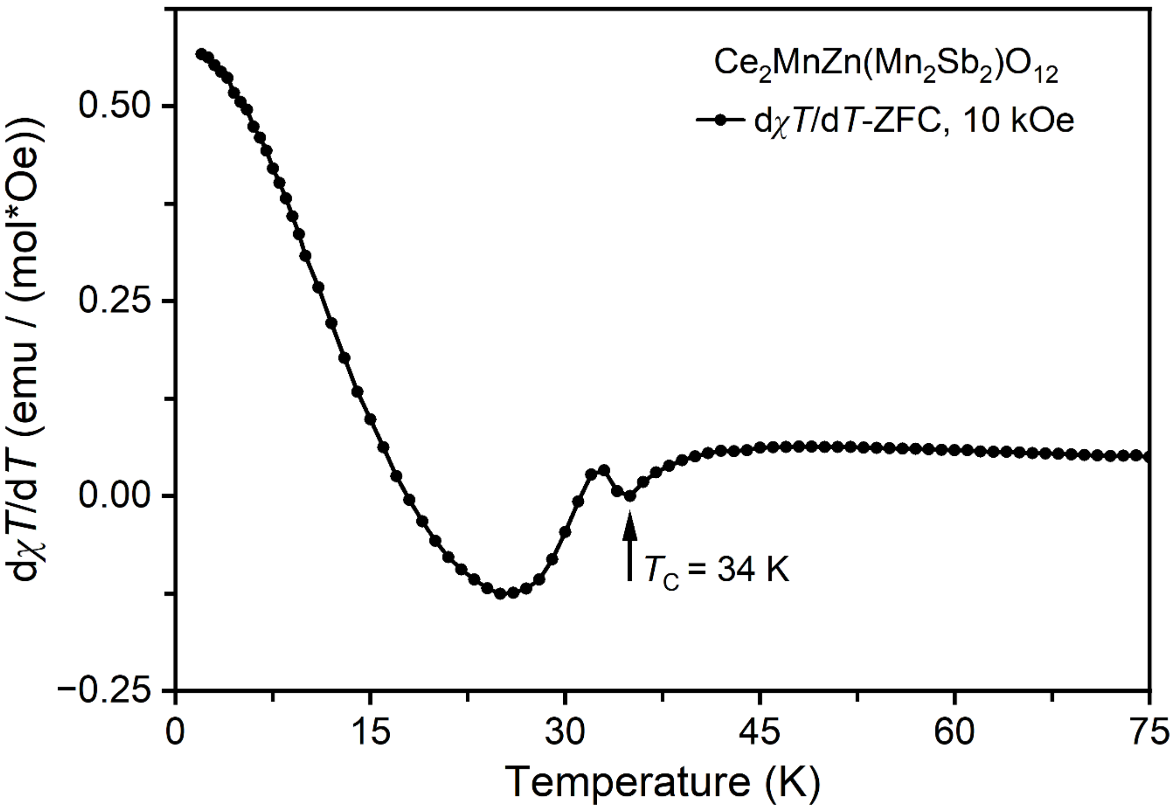


**Figure S4b.** ZFC d$\chi T$/d$T$ versus $T$ curve of $Ce_2MnZn(Mn_2Sb_2)O_{12}$ at $H$ = 10 kOe.

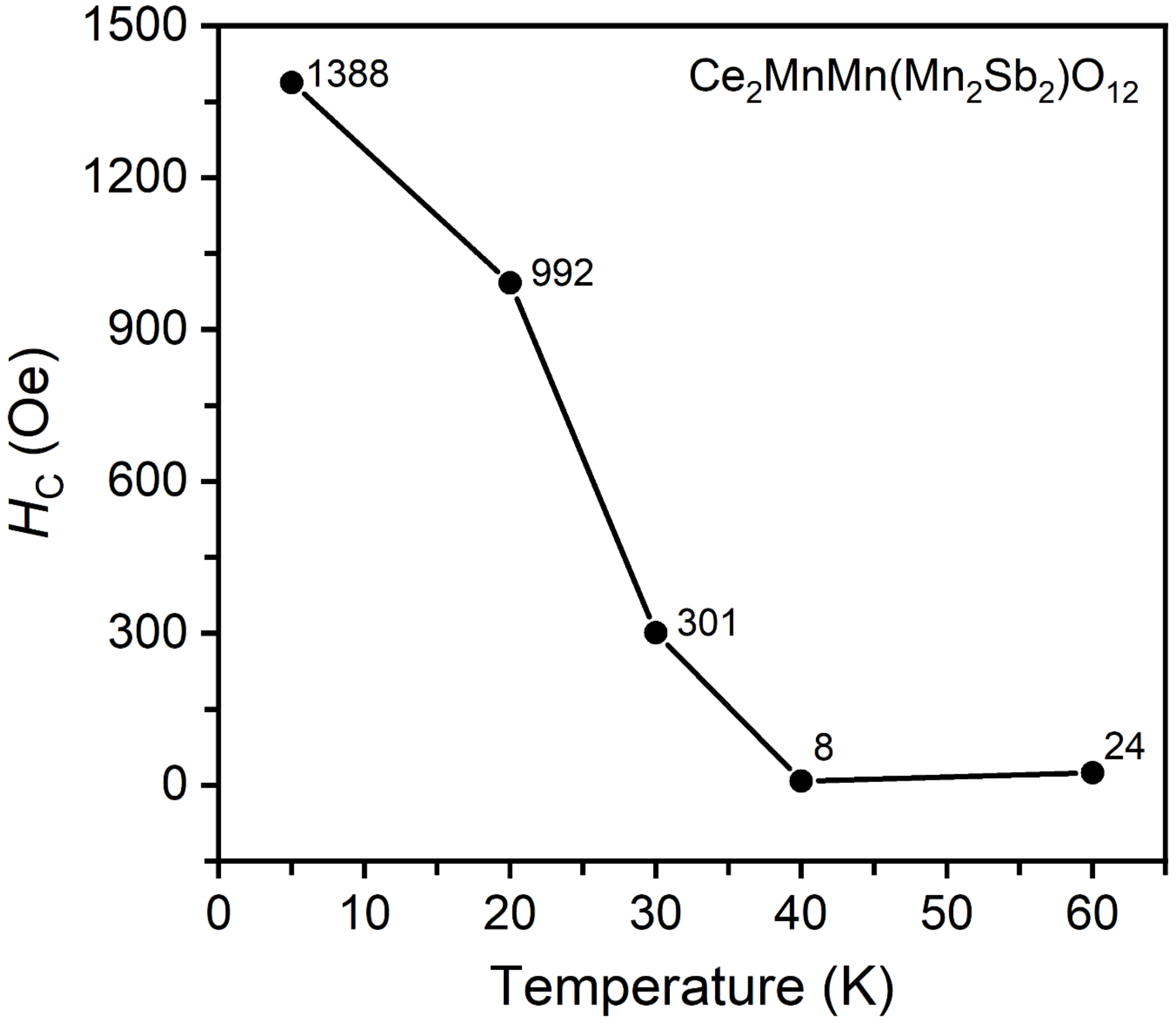


**Figure S5.** The coercive field ($H_C$) as a function of temperature for $Ce_2MnMn(Mn_2Sb_2)O_{12}$.

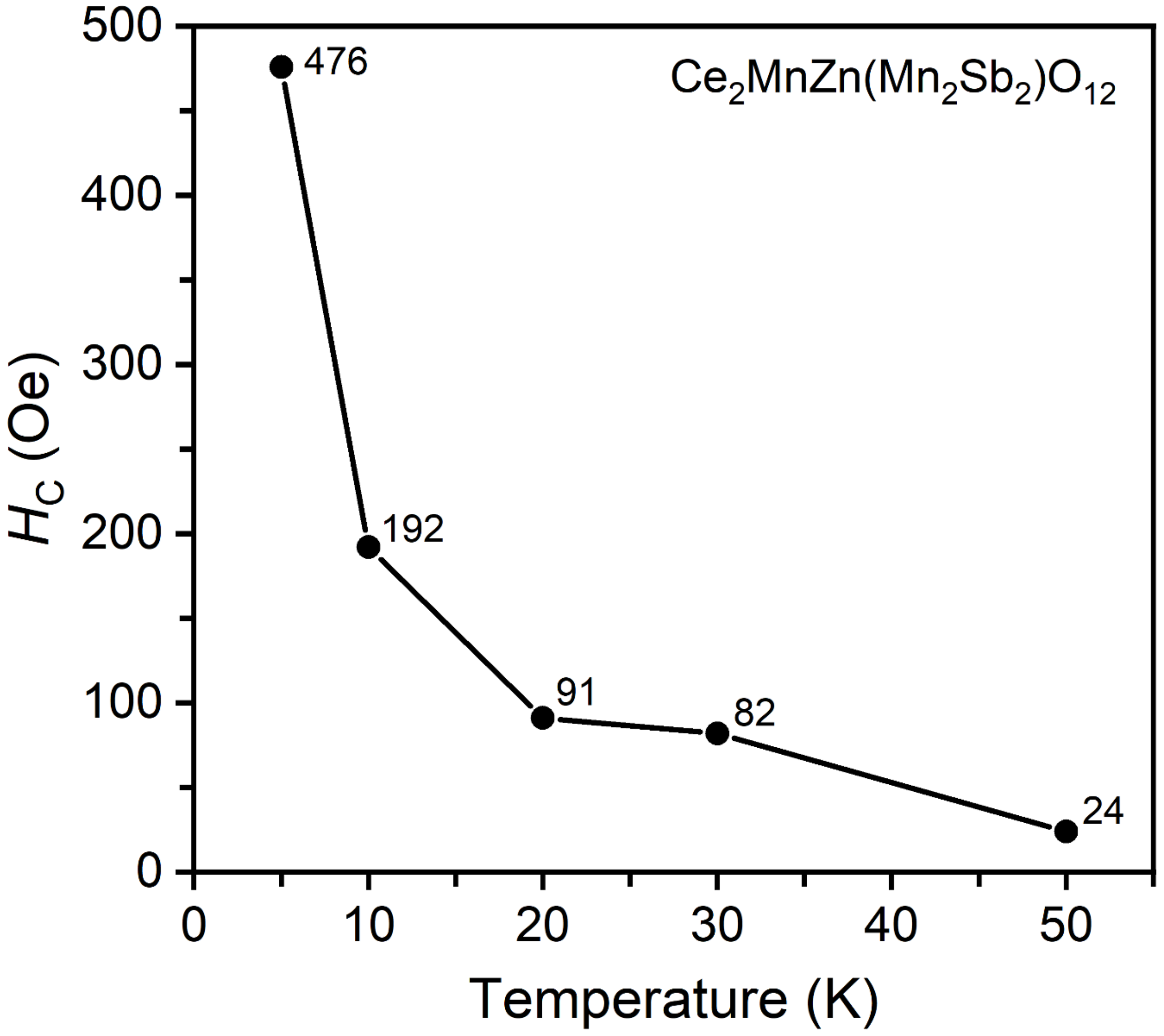


**Figure S6.** The coercive field ($H_C$) as a function of temperature for $Ce_2MnZn(Mn_2Sb_2)O_{12}$.

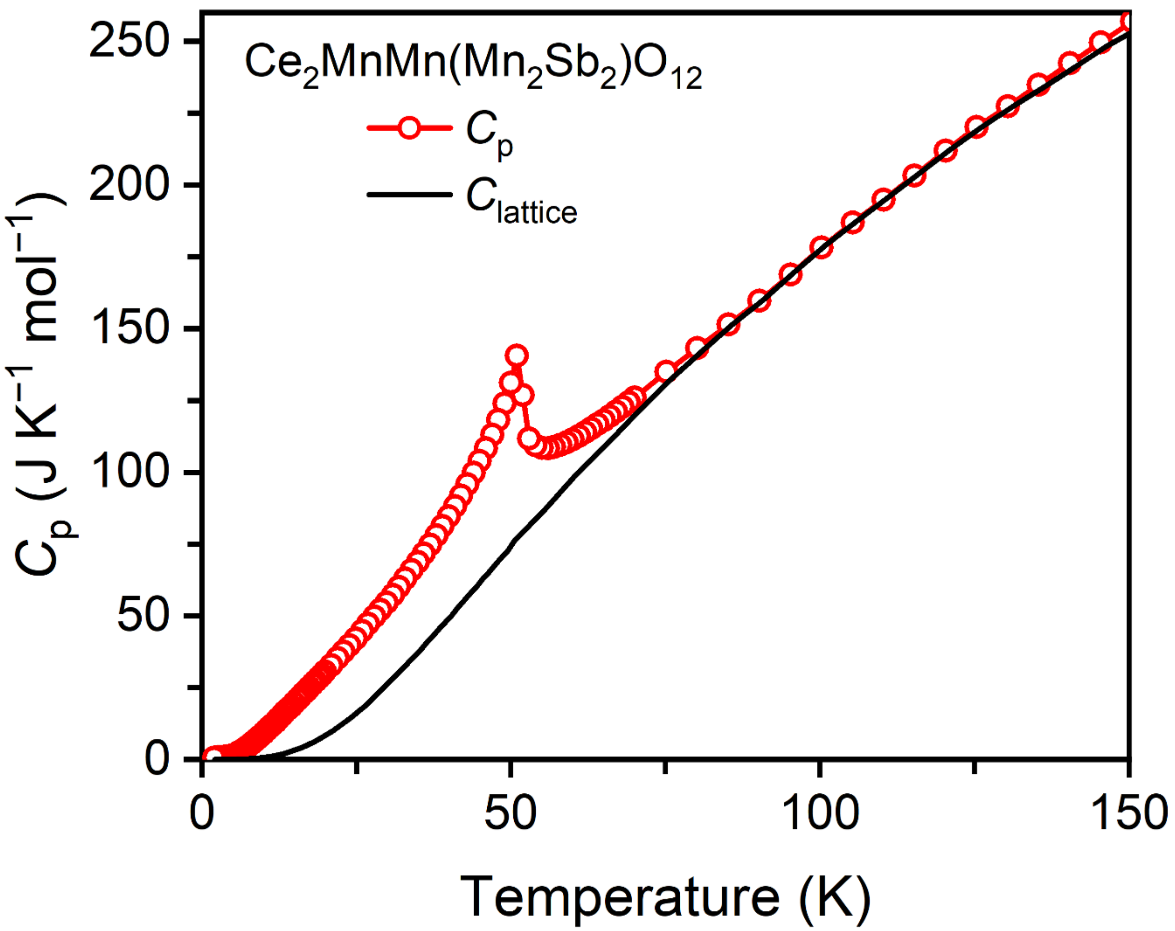


**Figure S7.** The heat capacity of $Ce_2MnMn(Mn_2Sb_2)O_{12}$ as a function of temperature. The black line shows the estimated lattice contribution (see the text).

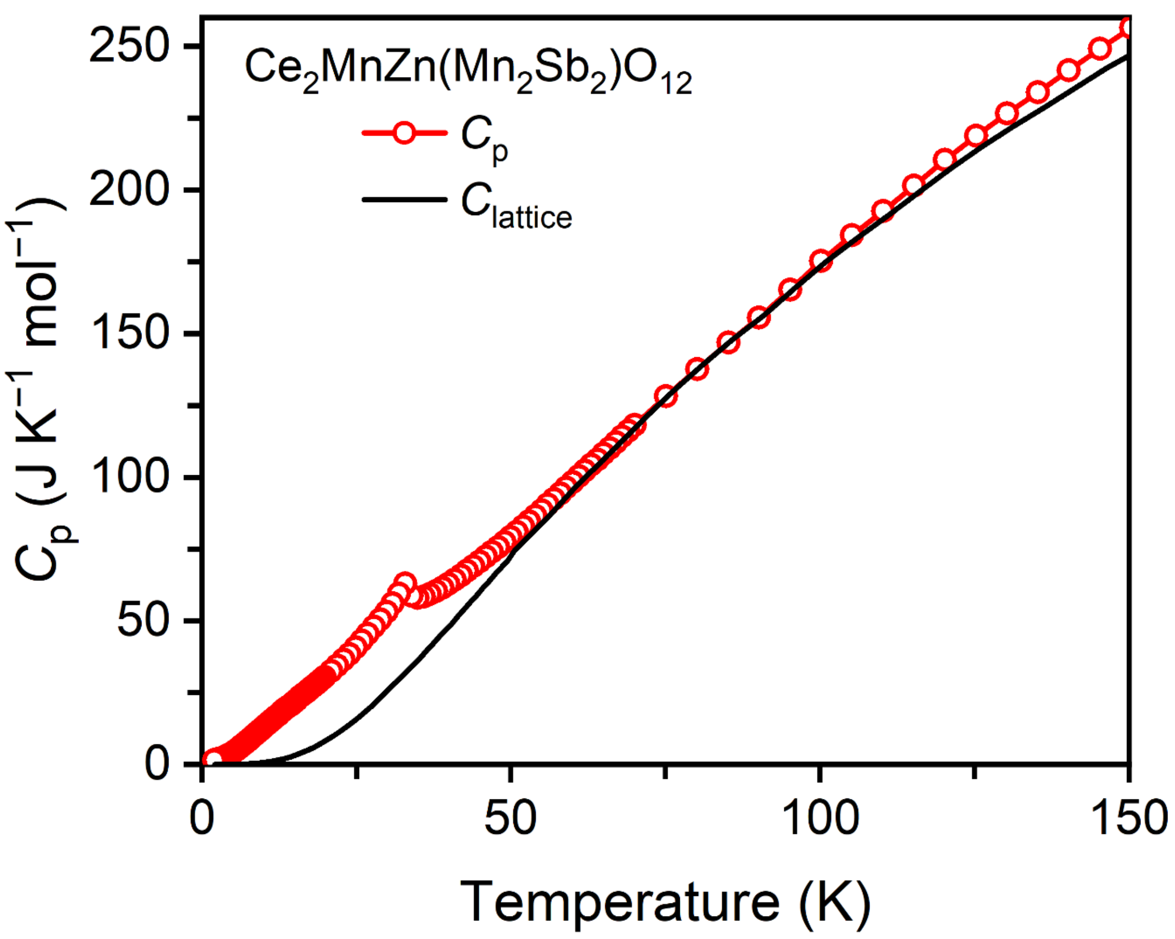


**Figure S8.** The heat capacity of $Ce_2MnZn(Mn_2Sb_2)O_{12}$ as a function of temperature. The black line shows the estimated lattice contribution (see the text).

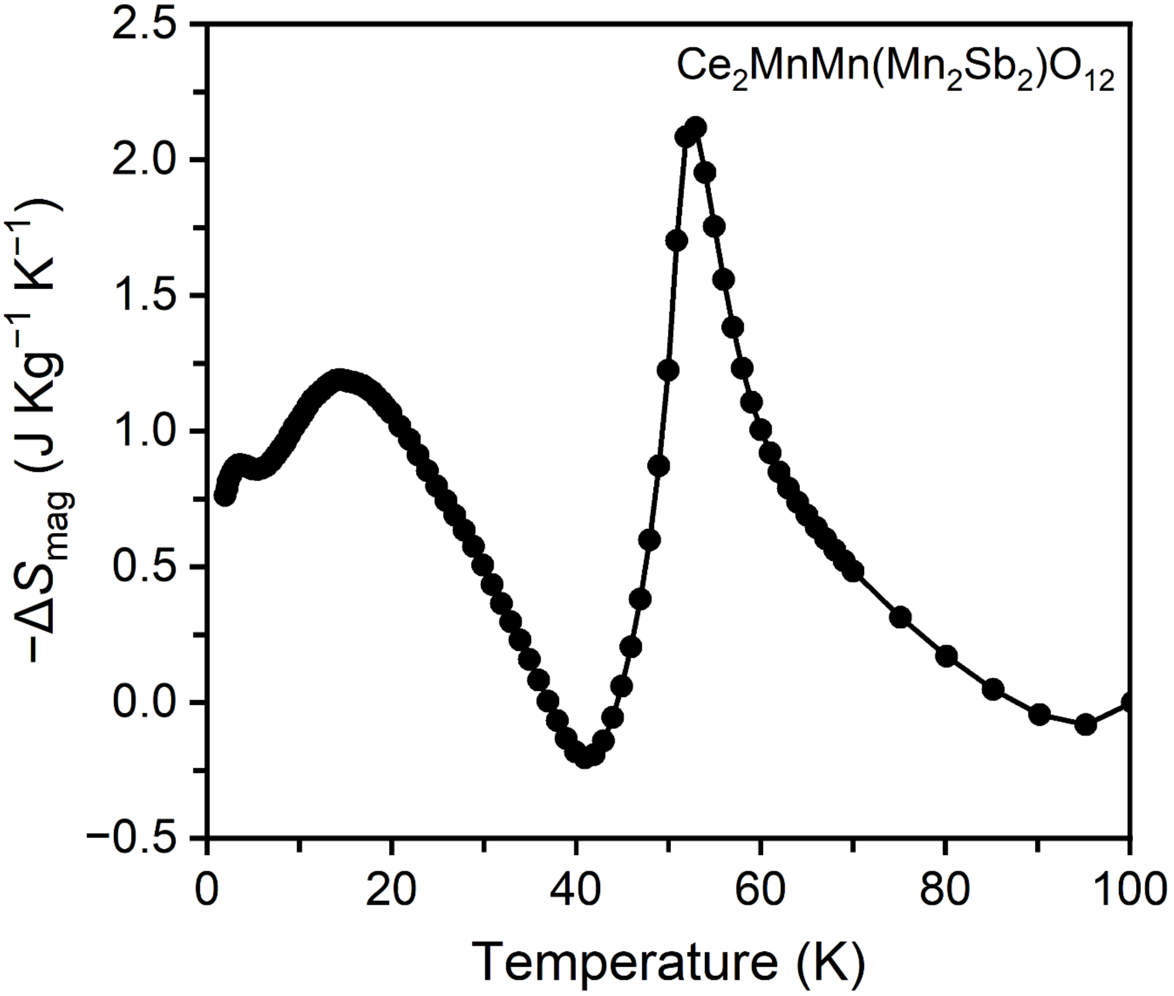


**Figure S9.** Temperature dependence of magnetic entropy change ($-\Delta S_{mag}$) under an applied magnetic field of $H$ = 90 kOe for $Ce_2MnMn(Mn_2Sb_2)O_{12}$.

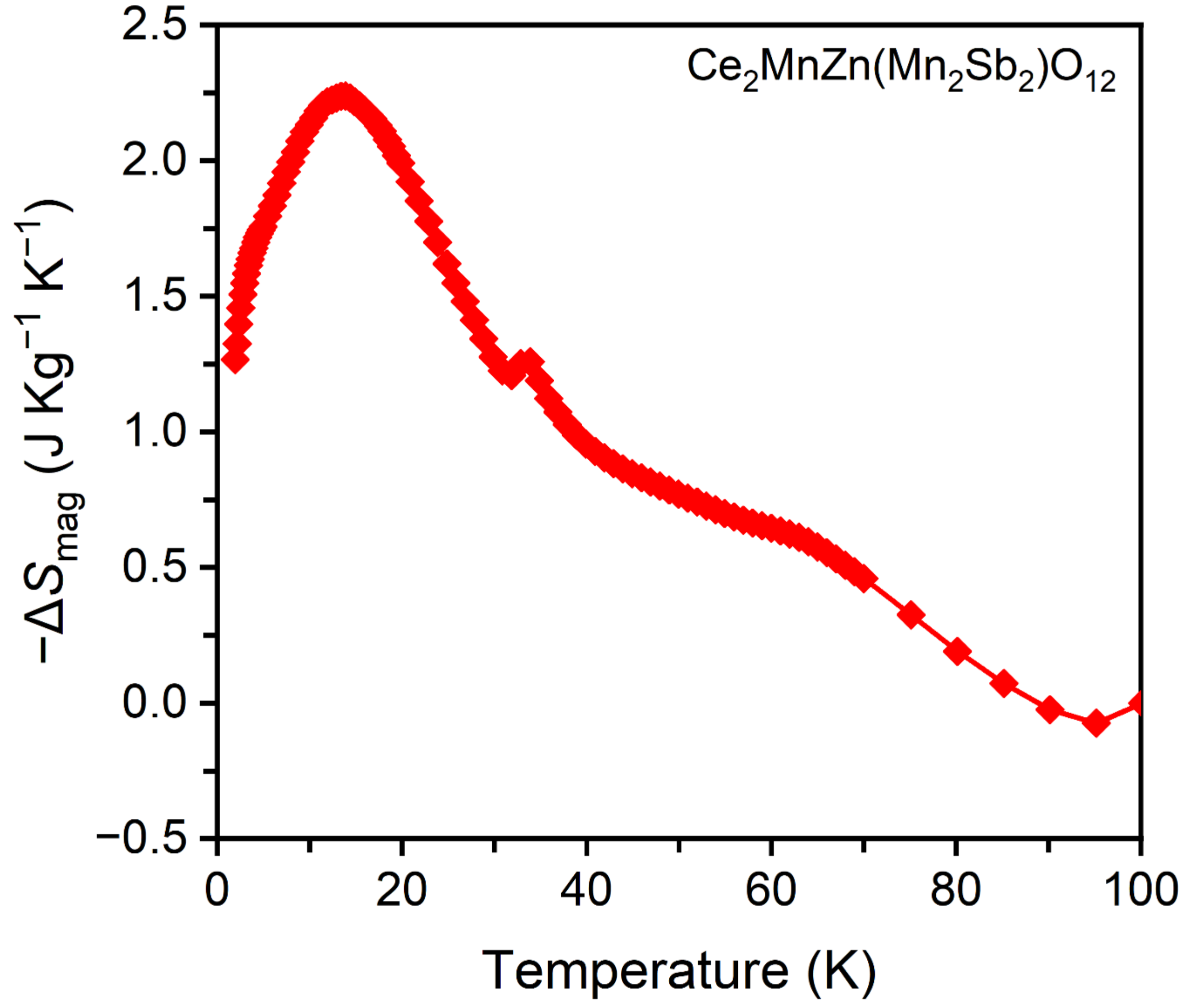


**Figure S10.** Temperature dependence of magnetic entropy change ($-\Delta S_{mag}$) under an applied magnetic field of $H = 90$ kOe for $Ce_2MnZn(Mn_2Sb_2)O_{12}$.

**Magnetic entropy change**

The relationship between magnetic entropy and temperature is used to study the magnetocaloric effect (MCE). Magnetic entropy change ($-\Delta S_{mag}$) is calculated based on the $C_p$ data by using the following equation[1]:

$$\Delta S_{\mathrm{mag}}(H_2, H_1) = \sum_{i=1}^{n} \frac{C_{H_2}(T_i) - C_{H_1}(T_i)}{T_i} \Delta T_i$$

where $C_{H_1}(T_i)$ and $C_{H_2}(T_i)$ are the values of $C_p$ data measured in the fields of $H_1 = 0$ Oe and $H_2 = 90$ kOe at $T_i$, respectively. The result of temperature dependence of $-\Delta S_{mag}$ is presented in **Figures S9–S10**, $-\Delta S_M$ increases after cooling the sample from the high temperature and reaches a maximum near $T_C$.

## REFERENCES


(1) Joshi, R. S.; Kumar, P. S. A. Magnetic Solid-State Materials. In *Comprehensive Inorganic Chemistry II: From Elements to Applications*, Reedijk, J., Poeppelmeier, K. Eds.; Vol. 4; Elsevier, 2013; pp 271–316.
