# Supplementary material for: Negative Magnetization Phenomena in A‑Site Columnar-Ordered Quadruple Perovskites Ce2MnM(Mn2Sb2)O12 with M = Mn and Zn
Source: Inorg Chem. 2025 May 16;64(21):10467–77. doi: 10.1021/acs.inorgchem.5c00653 (PMC12135034; doi:10.1021/acs.inorgchem.5c00653)
Supplement: Supplementary file 2 [file ic5c00653_si_002.pdf]

```
#==============================================================================

data_RIETAN_publ

#==============================================================================

_audit_creation_date  2025-04-01
_audit_creation_method  'Converted from *.lst using lst2cif'

#==============================================================================

# SUBMISSION DETAILS


_publ_contact_author_name         '?'
_publ_contact_author_address
;
;
_publ_contact_author_email        ?
_publ_contact_author_fax          '?'
_publ_contact_author_phone        '?'

_publ_requested_journal           '?'
_publ_requested_category          ?

_publ_contact_letter
;
;


#==============================================================================
```

PROCESSING SUMMARY (IUCr Office Use Only)

#_journal_date_recd_electronic

#_journal_date_to_coeditor

#_journal_date_from_coeditor

#_journal_date_accepted

#_journal_date_printers_first

#_journal_date_printers_final

#_journal_date_proofs_out

#_journal_date_proofs_in

#_journal_coeditor_name

#_journal_coeditor_code

#_journal_coeditor_notes

#_journal_techeditor_code

#_journal_paper_category

#_journal_compatibility_tag

#_journal_techeditor_notes

#_journal_coden_ASTM

#_journal_name_full

#_journal_year

#_journal_volume

#_journal_issue

#_journal_page_first

#_journal_page_last

#_journal_suppl_publ_number

#_journal_suppl_publ_pages

#==

```
# TITLE AND AUTHOR LIST

_publ_section_title
;
;

# The loop structure below should contain the names and addresses of all
# authors, in the required order of publication. Repeat as necessary.

loop_
  _publ_author_name
  _publ_author_address
  '?'
;
;


#==============================================================================

# TEXT

_publ_section_synopsis
;
;

_publ_section_abstract
;
;

_publ_section_comment
;
```

;

_publ_section_exptl_prep

;

;

_publ_section_exptl_refinement

;

;

_publ_section_references

;

;

_publ_section_figure_captions

;

;

_publ_section_acknowledgements

;

;

#==

CRYSTAL DATA

#--

data_RIETAN_phase_1

_pd_block_id

```
 '2025-04-01|PHASE_01|..creator_name..|..instr_name..'

_pd_phase_name                      Ce2MnMnMn2Sb2O12
_cell_length_a                      7.84545(1)
_cell_length_b                      7.84545(1)
_cell_length_c                      7.95529(2)
_cell_angle_alpha                   90.0
_cell_angle_beta                    90.0
_cell_angle_gamma                   90.0
_cell_volume                        489.657(1)
_cell_formula_units                 2
_symmetry_cell_setting              tetragonal
_symmetry_space_group_name_H-M      'P 42/n'
_symmetry_Int_Tables_number         86

loop_
  _symmetry_equiv_pos_site_id
  _symmetry_equiv_pos_as_xyz
    1   x,y,z
    2   -x+1/2,-y+1/2,z
    3   -y,x+1/2,z+1/2
    4   y+1/2,-x,z+1/2
    5   -x,-y,-z
    6   x+1/2,y+1/2,-z
    7   y,-x+1/2,-z+1/2
    8   -y+1/2,x,-z+1/2

loop_
  _atom_site_label
  _atom_site_symmetry_multiplicity
  _atom_site_occupancy
```

```
  _atom_site_fract_x
  _atom_site_fract_y
  _atom_site_fract_z
  _atom_site_thermal_displace_type
  _atom_site_B_iso_or_equiv
  _atom_site_type_symbol
  Ce      4 0.928(4)   0.25      0.75      0.77609(6) Biso  0.580(11) Ce
  Mn      4 0.072(4)   0.25      0.75      0.77609(6) Biso  0.580(11) Mn
  Mn1-SQ   2 1.0      0.25      0.25      0.75      Biso  1.17(9)   Mn
  Mn2-T    2 1.0      0.75      0.75      0.75      Biso  0.61(8)   Mn
  Mn3-O    4 1.0      0.0       0.5       0.5       Biso  0.65(2)   Mn
  Sb-O     4 1.0      0.0       0.0       0.5       Biso  0.375(9)  Sb
  O1      8 1.0      -0.0461(9)   0.5738(9)   0.2345(7)  Biso  0.52(11)  O
  O2      8 1.0      -0.2348(12) -0.0430(7)   0.5831(6)  Biso  0.90(12)  O
  O3      8 1.0      -0.2587(10)  0.0671(6)  -0.0319(6)  Biso  0.51(9)   O
#----------------------------------------------------------------------
data_RIETAN_phase_2

_pd_block_id
    '2025-04-01|PHASE_02|..creator_name..|..instr_name..'

_pd_phase_name                    CeO2
_cell_length_a                 5.41092
_cell_length_b                 5.41092
_cell_length_c                 5.41092
_cell_angle_alpha                90.0
_cell_angle_beta                90.0
_cell_angle_gamma                90.0
_cell_volume                   158.4212
_cell_formula_units                ?
_symmetry_cell_setting             cubic
```

_symmetry_space_group_name_H-M 'F m -3 m'

_symmetry_Int_Tables_number 225

```
loop_
  _symmetry_equiv_pos_site_id
  _symmetry_equiv_pos_as_xyz
    1  x,y,z
    2  -x,-y,z
    3  -x,y,-z
    4  x,-y,-z
    5  z,x,y
    6  z,-x,-y
    7  -z,-x,y
    8  -z,x,-y
    9  y,z,x
   10  -y,z,-x
   11  y,-z,-x
   12  -y,-z,x
   13  y,x,-z
   14  -y,-x,-z
   15  y,-x,z
   16  -y,x,z
   17  x,z,-y
   18  -x,z,y
   19  -x,-z,-y
   20  x,-z,y
   21  z,y,-x
   22  z,-y,x
   23  -z,y,x
   24  -z,-y,-x
   25  -x,-y,-z
```

26 x,y,-z

27 x,-y,z

28 -x,y,z

29 -z,-x,-y

30 -z,x,y

31 z,x,-y

32 z,-x,y

33 -y,-z,-x

34 y,-z,x

35 -y,z,x

36 y,z,-x

37 -y,-x,z

38 y,x,z

39 -y,x,-z

40 y,-x,-z

41 -x,-z,y

42 x,-z,-y

43 x,z,y

44 -x,z,-y

45 -z,-y,x

46 -z,y,-x

47 z,-y,-x

48 z,y,x

49 x+1/2,y+1/2,z

50 -x+1/2,-y+1/2,z

51 -x+1/2,y+1/2,-z

52 x+1/2,-y+1/2,-z

53 z+1/2,x+1/2,y

54 z+1/2,-x+1/2,-y

55 -z+1/2,-x+1/2,y

56 -z+1/2,x+1/2,-y

57 y+1/2,z+1/2,x

58 -y+1/2,z+1/2,-x

59 y+1/2,-z+1/2,-x

60 -y+1/2,-z+1/2,x

61 y+1/2,x+1/2,-z

62 -y+1/2,-x+1/2,-z

63 y+1/2,-x+1/2,z

64 -y+1/2,x+1/2,z

65 x+1/2,z+1/2,-y

66 -x+1/2,z+1/2,y

67 -x+1/2,-z+1/2,-y

68 x+1/2,-z+1/2,y

69 z+1/2,y+1/2,-x

70 z+1/2,-y+1/2,x

71 -z+1/2,y+1/2,x

72 -z+1/2,-y+1/2,-x

73 -x+1/2,-y+1/2,-z

74 x+1/2,y+1/2,-z

75 x+1/2,-y+1/2,z

76 -x+1/2,y+1/2,z

77 -z+1/2,-x+1/2,-y

78 -z+1/2,x+1/2,y

79 z+1/2,x+1/2,-y

80 z+1/2,-x+1/2,y

81 -y+1/2,-z+1/2,-x

82 y+1/2,-z+1/2,x

83 -y+1/2,z+1/2,x

84 y+1/2,z+1/2,-x

85 -y+1/2,-x+1/2,z

86 y+1/2,x+1/2,z

87 -y+1/2,x+1/2,-z

88 y+1/2,-x+1/2,-z

89 -x+1/2,-z+1/2,y

90 x+1/2,-z+1/2,-y

91 x+1/2,z+1/2,y

92 -x+1/2,z+1/2,-y

93 -z+1/2,-y+1/2,x

94 -z+1/2,y+1/2,-x

95 z+1/2,-y+1/2,-x

96 z+1/2,y+1/2,x

97 x+1/2,y,z+1/2

98 -x+1/2,-y,z+1/2

99 -x+1/2,y,-z+1/2

100 x+1/2,-y,-z+1/2

101 z+1/2,x,y+1/2

102 z+1/2,-x,-y+1/2

103 -z+1/2,-x,y+1/2

104 -z+1/2,x,-y+1/2

105 y+1/2,z,x+1/2

106 -y+1/2,z,-x+1/2

107 y+1/2,-z,-x+1/2

108 -y+1/2,-z,x+1/2

109 y+1/2,x,-z+1/2

110 -y+1/2,-x,-z+1/2

111 y+1/2,-x,z+1/2

112 -y+1/2,x,z+1/2

113 x+1/2,z,-y+1/2

114 -x+1/2,z,y+1/2

115 -x+1/2,-z,-y+1/2

116 x+1/2,-z,y+1/2

117 z+1/2,y,-x+1/2

118 z+1/2,-y,x+1/2

119 -z+1/2,y,x+1/2

120 -z+1/2,-y,-x+1/2

121 -x+1/2,-y,-z+1/2

122 x+1/2,y,-z+1/2

123 x+1/2,-y,z+1/2

124 -x+1/2,y,z+1/2

125 -z+1/2,-x,-y+1/2

126 -z+1/2,x,y+1/2

127 z+1/2,x,-y+1/2

128 z+1/2,-x,y+1/2

129 -y+1/2,-z,-x+1/2

130 y+1/2,-z,x+1/2

131 -y+1/2,z,x+1/2

132 y+1/2,z,-x+1/2

133 -y+1/2,-x,z+1/2

134 y+1/2,x,z+1/2

135 -y+1/2,x,-z+1/2

136 y+1/2,-x,-z+1/2

137 -x+1/2,-z,y+1/2

138 x+1/2,-z,-y+1/2

139 x+1/2,z,y+1/2

140 -x+1/2,z,-y+1/2

141 -z+1/2,-y,x+1/2

142 -z+1/2,y,-x+1/2

143 z+1/2,-y,-x+1/2

144 z+1/2,y,x+1/2

145 x,y+1/2,z+1/2

146 -x,-y+1/2,z+1/2

147 -x,y+1/2,-z+1/2

148 x,-y+1/2,-z+1/2

149 z,x+1/2,y+1/2

150 z,-x+1/2,-y+1/2

151 -z,-x+1/2,y+1/2

152 -z,x+1/2,-y+1/2

153 y,z+1/2,x+1/2

154 -y,z+1/2,-x+1/2

155 y,-z+1/2,-x+1/2

156 -y,-z+1/2,x+1/2

157 y,x+1/2,-z+1/2

158 -y,-x+1/2,-z+1/2

159 y,-x+1/2,z+1/2

160 -y,x+1/2,z+1/2

161 x,z+1/2,-y+1/2

162 -x,z+1/2,y+1/2

163 -x,-z+1/2,-y+1/2

164 x,-z+1/2,y+1/2

165 z,y+1/2,-x+1/2

166 z,-y+1/2,x+1/2

167 -z,y+1/2,x+1/2

168 -z,-y+1/2,-x+1/2

169 -x,-y+1/2,-z+1/2

170 x,y+1/2,-z+1/2

171 x,-y+1/2,z+1/2

172 -x,y+1/2,z+1/2

173 -z,-x+1/2,-y+1/2

174 -z,x+1/2,y+1/2

175 z,x+1/2,-y+1/2

176 z,-x+1/2,y+1/2

177 -y,-z+1/2,-x+1/2

178 y,-z+1/2,x+1/2

179 -y,z+1/2,x+1/2

180 y,z+1/2,-x+1/2

```
  181   -y,-x+1/2,z+1/2
  182   y,x+1/2,z+1/2
  183   -y,x+1/2,-z+1/2
  184   y,-x+1/2,-z+1/2
  185   -x,-z+1/2,y+1/2
  186   x,-z+1/2,-y+1/2
  187   x,z+1/2,y+1/2
  188   -x,z+1/2,-y+1/2
  189   -z,-y+1/2,x+1/2
  190   -z,y+1/2,-x+1/2
  191   z,-y+1/2,-x+1/2
  192   z,y+1/2,x+1/2

loop_
  _atom_site_label
  _atom_site_symmetry_multiplicity
  _atom_site_occupancy
  _atom_site_fract_x
  _atom_site_fract_y
  _atom_site_fract_z
  _atom_site_thermal_displace_type
  _atom_site_B_iso_or_equiv
  _atom_site_type_symbol
  Ce51     4 1.0       0.0        0.0        0.0       Biso  0.5      Ce
  O511     8 1.0       0.25       0.25       0.25      Biso  1.0      O
#------------------------------------------------------------------------
data_RIETAN_phase_3

_pd_block_id
      '2025-04-01|PHASE_03|..creator_name..|..instr_name..'
```

```
_pd_phase_name                    Sb2O4+d, pyrochlore
_cell_length_a                    10.26330
_cell_length_b                    10.26330
_cell_length_c                    10.26330
_cell_angle_alpha                 90.0
_cell_angle_beta                  90.0
_cell_angle_gamma                 90.0
_cell_volume                      1081.0879
_cell_formula_units               ?
_symmetry_cell_setting            cubic
_symmetry_space_group_name_H-M    'F d -3 m'
_symmetry_Int_Tables_number       227

loop_
  _symmetry_equiv_pos_site_id
  _symmetry_equiv_pos_as_xyz
    1  x,y,z
    2  -x+3/4,-y+1/4,z+1/2
    3  -x+1/4,y+1/2,-z+3/4
    4  x+1/2,-y+3/4,-z+1/4
    5  z,x,y
    6  z+1/2,-x+3/4,-y+1/4
    7  -z+3/4,-x+1/4,y+1/2
    8  -z+1/4,x+1/2,-y+3/4
    9  y,z,x
   10  -y+1/4,z+1/2,-x+3/4
   11  y+1/2,-z+3/4,-x+1/4
   12  -y+3/4,-z+1/4,x+1/2
   13  y+3/4,x+1/4,-z+1/2
   14  -y,-x,-z
   15  y+1/4,-x+1/2,z+3/4
```

16 -y+1/2,x+3/4,z+1/4

17 x+3/4,z+1/4,-y+1/2

18 -x+1/2,z+3/4,y+1/4

19 -x,-z,-y

20 x+1/4,-z+1/2,y+3/4

21 z+3/4,y+1/4,-x+1/2

22 z+1/4,-y+1/2,x+3/4

23 -z+1/2,y+3/4,x+1/4

24 -z,-y,-x

25 -x,-y,-z

26 x+1/4,y+3/4,-z+1/2

27 x+3/4,-y+1/2,z+1/4

28 -x+1/2,y+1/4,z+3/4

29 -z,-x,-y

30 -z+1/2,x+1/4,y+3/4

31 z+1/4,x+3/4,-y+1/2

32 z+3/4,-x+1/2,y+1/4

33 -y,-z,-x

34 y+3/4,-z+1/2,x+1/4

35 -y+1/2,z+1/4,x+3/4

36 y+1/4,z+3/4,-x+1/2

37 -y+1/4,-x+3/4,z+1/2

38 y,x,z

39 -y+3/4,x+1/2,-z+1/4

40 y+1/2,-x+1/4,-z+3/4

41 -x+1/4,-z+3/4,y+1/2

42 x+1/2,-z+1/4,-y+3/4

43 x,z,y

44 -x+3/4,z+1/2,-y+1/4

45 -z+1/4,-y+3/4,x+1/2

46 -z+3/4,y+1/2,-x+1/4

47 z+1/2,-y+1/4,-x+3/4

48 z,y,x

49 x+1/2,y+1/2,z

50 -x+1/4,-y+3/4,z+1/2

51 -x+3/4,y,-z+3/4

52 x,-y+1/4,-z+1/4

53 z+1/2,x+1/2,y

54 z,-x+1/4,-y+1/4

55 -z+1/4,-x+3/4,y+1/2

56 -z+3/4,x,-y+3/4

57 y+1/2,z+1/2,x

58 -y+3/4,z,-x+3/4

59 y,-z+1/4,-x+1/4

60 -y+1/4,-z+3/4,x+1/2

61 y+1/4,x+3/4,-z+1/2

62 -y+1/2,-x+1/2,-z

63 y+3/4,-x,z+3/4

64 -y,x+1/4,z+1/4

65 x+1/4,z+3/4,-y+1/2

66 -x,z+1/4,y+1/4

67 -x+1/2,-z+1/2,-y

68 x+3/4,-z,y+3/4

69 z+1/4,y+3/4,-x+1/2

70 z+3/4,-y,x+3/4

71 -z,y+1/4,x+1/4

72 -z+1/2,-y+1/2,-x

73 -x+1/2,-y+1/2,-z

74 x+3/4,y+1/4,-z+1/2

75 x+1/4,-y,z+1/4

76 -x,y+3/4,z+3/4

77 -z+1/2,-x+1/2,-y

78 -z,x+3/4,y+3/4

79 z+3/4,x+1/4,-y+1/2

80 z+1/4,-x,y+1/4

81 -y+1/2,-z+1/2,-x

82 y+1/4,-z,x+1/4

83 -y,z+3/4,x+3/4

84 y+3/4,z+1/4,-x+1/2

85 -y+3/4,-x+1/4,z+1/2

86 y+1/2,x+1/2,z

87 -y+1/4,x,-z+1/4

88 y,-x+3/4,-z+3/4

89 -x+3/4,-z+1/4,y+1/2

90 x,-z+3/4,-y+3/4

91 x+1/2,z+1/2,y

92 -x+1/4,z,-y+1/4

93 -z+3/4,-y+1/4,x+1/2

94 -z+1/4,y,-x+1/4

95 z,-y+3/4,-x+3/4

96 z+1/2,y+1/2,x

97 x+1/2,y,z+1/2

98 -x+1/4,-y+1/4,z

99 -x+3/4,y+1/2,-z+1/4

100 x,-y+3/4,-z+3/4

101 z+1/2,x,y+1/2

102 z,-x+3/4,-y+3/4

103 -z+1/4,-x+1/4,y

104 -z+3/4,x+1/2,-y+1/4

105 y+1/2,z,x+1/2

106 -y+3/4,z+1/2,-x+1/4

107 y,-z+3/4,-x+3/4

108 -y+1/4,-z+1/4,x

109 y+1/4,x+1/4,-z

110 -y+1/2,-x,-z+1/2

111 y+3/4,-x+1/2,z+1/4

112 -y,x+3/4,z+3/4

113 x+1/4,z+1/4,-y

114 -x,z+3/4,y+3/4

115 -x+1/2,-z,-y+1/2

116 x+3/4,-z+1/2,y+1/4

117 z+1/4,y+1/4,-x

118 z+3/4,-y+1/2,x+1/4

119 -z,y+3/4,x+3/4

120 -z+1/2,-y,-x+1/2

121 -x+1/2,-y,-z+1/2

122 x+3/4,y+3/4,-z

123 x+1/4,-y+1/2,z+3/4

124 -x,y+1/4,z+1/4

125 -z+1/2,-x,-y+1/2

126 -z,x+1/4,y+1/4

127 z+3/4,x+3/4,-y

128 z+1/4,-x+1/2,y+3/4

129 -y+1/2,-z,-x+1/2

130 y+1/4,-z+1/2,x+3/4

131 -y,z+1/4,x+1/4

132 y+3/4,z+3/4,-x

133 -y+3/4,-x+3/4,z

134 y+1/2,x,z+1/2

135 -y+1/4,x+1/2,-z+3/4

136 y,-x+1/4,-z+1/4

137 -x+3/4,-z+3/4,y

138 x,-z+1/4,-y+1/4

139 x+1/2,z,y+1/2

140 -x+1/4,z+1/2,-y+3/4

141 -z+3/4,-y+3/4,x

142 -z+1/4,y+1/2,-x+3/4

143 z,-y+1/4,-x+1/4

144 z+1/2,y,x+1/2

145 x,y+1/2,z+1/2

146 -x+3/4,-y+3/4,z

147 -x+1/4,y,-z+1/4

148 x+1/2,-y+1/4,-z+3/4

149 z,x+1/2,y+1/2

150 z+1/2,-x+1/4,-y+3/4

151 -z+3/4,-x+3/4,y

152 -z+1/4,x,-y+1/4

153 y,z+1/2,x+1/2

154 -y+1/4,z,-x+1/4

155 y+1/2,-z+1/4,-x+3/4

156 -y+3/4,-z+3/4,x

157 y+3/4,x+3/4,-z

158 -y,-x+1/2,-z+1/2

159 y+1/4,-x,z+1/4

160 -y+1/2,x+1/4,z+3/4

161 x+3/4,z+3/4,-y

162 -x+1/2,z+1/4,y+3/4

163 -x,-z+1/2,-y+1/2

164 x+1/4,-z,y+1/4

165 z+3/4,y+3/4,-x

166 z+1/4,-y,x+1/4

167 -z+1/2,y+1/4,x+3/4

168 -z,-y+1/2,-x+1/2

169 -x,-y+1/2,-z+1/2

170 x+1/4,y+1/4,-z

171 x+3/4,-y,z+3/4

172 -x+1/2,y+3/4,z+1/4

173 -z,-x+1/2,-y+1/2

174 -z+1/2,x+3/4,y+1/4

175 z+1/4,x+1/4,-y

176 z+3/4,-x,y+3/4

177 -y,-z+1/2,-x+1/2

178 y+3/4,-z,x+3/4

179 -y+1/2,z+3/4,x+1/4

180 y+1/4,z+1/4,-x

181 -y+1/4,-x+1/4,z

182 y,x+1/2,z+1/2

183 -y+3/4,x,-z+3/4

184 y+1/2,-x+3/4,-z+1/4

185 -x+1/4,-z+1/4,y

186 x+1/2,-z+3/4,-y+1/4

187 x,z+1/2,y+1/2

188 -x+3/4,z,-y+3/4

189 -z+1/4,-y+1/4,x

190 -z+3/4,y,-x+3/4

191 z+1/2,-y+3/4,-x+1/4

192 z,y+1/2,x+1/2

loop_

_atom_site_label

_atom_site_symmetry_multiplicity

_atom_site_occupancy

_atom_site_fract_x

_atom_site_fract_y

_atom_site_fract_z

_atom_site_thermal_displace_type

```
  _atom_site_B_iso_or_equiv
  _atom_site_type_symbol
  Sb-1    16 1.0       0.5        0.5        0.5       Biso  0.49     Sb
  Sb-2    16 1.0       0.0        0.0        0.0       Biso  0.49     Sb
  O-1     8 1.0       0.375      0.375      0.375      Biso  0.36     O
  O-2    48 1.0       0.3265     0.125      0.125      Biso  0.36     O
#------------------------------------------------------------------------
data_RIETAN_phase_4

_pd_block_id
    '2025-04-01|PHASE_04|..creator_name..|..instr_name..'

_pd_phase_name                     La3Mn2Sb3O14 R-3m
_cell_length_a                  7.42618
_cell_length_b                  7.42618
_cell_length_c                  17.63940
_cell_angle_alpha                  90.0
_cell_angle_beta                  90.0
_cell_angle_gamma                   120.0
_cell_volume                    842.4524
_cell_formula_units                  ?
_symmetry_cell_setting               trigonal
_symmetry_space_group_name_H-M        'R -3 m'
_symmetry_Int_Tables_number          166

loop_
  _symmetry_equiv_pos_site_id
  _symmetry_equiv_pos_as_xyz
    1   x,y,z
    2   -y,x-y,z
    3   -x+y,-x,z
```

4 y,x,-z

5 x-y,-y,-z

6 -x,-x+y,-z

7 -x,-y,-z

8 y,-x+y,-z

9 x-y,x,-z

10 -y,-x,z

11 -x+y,y,z

12 x,x-y,z

13 x+2/3,y+1/3,z+1/3

14 -y+2/3,x-y+1/3,z+1/3

15 -x+y+2/3,-x+1/3,z+1/3

16 y+2/3,x+1/3,-z+1/3

17 x-y+2/3,-y+1/3,-z+1/3

18 -x+2/3,-x+y+1/3,-z+1/3

19 -x+2/3,-y+1/3,-z+1/3

20 y+2/3,-x+y+1/3,-z+1/3

21 x-y+2/3,x+1/3,-z+1/3

22 -y+2/3,-x+1/3,z+1/3

23 -x+y+2/3,y+1/3,z+1/3

24 x+2/3,x-y+1/3,z+1/3

25 x+1/3,y+2/3,z+2/3

26 -y+1/3,x-y+2/3,z+2/3

27 -x+y+1/3,-x+2/3,z+2/3

28 y+1/3,x+2/3,-z+2/3

29 x-y+1/3,-y+2/3,-z+2/3

30 -x+1/3,-x+y+2/3,-z+2/3

31 -x+1/3,-y+2/3,-z+2/3

32 y+1/3,-x+y+2/3,-z+2/3

33 x-y+1/3,x+2/3,-z+2/3

34 -y+1/3,-x+2/3,z+2/3

```
   35   -x+y+1/3,y+2/3,z+2/3
   36   x+1/3,x-y+2/3,z+2/3

loop_
  _atom_site_label
  _atom_site_symmetry_multiplicity
  _atom_site_occupancy
  _atom_site_fract_x
  _atom_site_fract_y
  _atom_site_fract_z
  _atom_site_thermal_displace_type
  _atom_site_B_iso_or_equiv
  _atom_site_type_symbol
  Mn-1a    3 1.0       0.0        0.0        0.5       Biso  0.5      Mn
  Ce-2a    9 1.0       0.5        0.0        0.5       Biso  0.5      Ce
  Mn-2a    3 1.0       0.0        0.0        0.0       Biso  0.5      Mn
  Sb-2a    9 1.0       0.5        0.0        0.0       Biso  0.5      Sb
  O-1n    18 1.0       0.4762     0.5238     0.1116    Biso  0.8      O
  O-2n    18 1.0       0.4664     0.5336     0.3498    Biso  0.8      O
  O-3n     6 1.0       0.0        0.0        0.114     Biso  0.8      O

#=========================================================================

# POWDER SPECIMEN AND EXPERIMENTAL DATA

data_RIETAN_p_01

_pd_block_id
    '2025-04-01|POWSET_01|..creator_name..|..instr_name..'
_pd_meas_datetime_initialed           ?
_pd_meas_info_author_name             "?"
```

_pd_meas_info_author_email ?

_pd_meas_info_author_address

;

;

_pd_calc_method "Rietveld Refinement"

_diffrn_ambient_temperature ?

_diffrn_ambient_environment ?

_diffrn_source '?'

_diffrn_source_target ?

_diffrn_source_type ?

_diffrn_measurement_device_type '?'

_diffrn_detector '?'

_diffrn_detector_type ?

_pd_meas_scan_method step

_pd_meas_special_details

;

;

_diffrn_radiation_type 'synchrotron X-ray'

_diffrn_radiation_wavelength 0.61974

_diffrn_radiation_monochromator none

_pd_meas_2theta_range_min 5.004

_pd_meas_2theta_range_max 71.250

_pd_meas_2theta_range_inc 0.006

_pd_meas_number_of_points 11042

#==

REFINEMENT DATA

```
_pd_proc_ls_special_details
;
;

_pd_proc_ls_profile_function     ?
_pd_proc_ls_background_function   '?'
_pd_proc_ls_pref_orient_corr
;
;

_pd_proc_ls_prof_R_factor        0.0642
_pd_proc_ls_prof_wR_factor       0.0849
_pd_proc_ls_prof_wR_expected     0.016
_refine_special_details
;
;

_refine_ls_structure_factor_coef    Inet
_refine_ls_matrix_type             ?
_refine_ls_weighting_sheme         '?'
_refine_ls_hydrogen_treatment      noref
_refine_ls_extinction_method       none
_refine_ls_extinction_coef         ?
_refine_ls_number_parameters       ?
_refine_ls_number_constraints      3
_refine_ls_goodness_of_fit_all     5.20


#--eof--eof--eof--eof--eof--eof--eof--eof--eof--eof--eof--eof--eof--eof--eof--#
```
