# Supplementary material for: Negative Magnetization Phenomena in A‑Site Columnar-Ordered Quadruple Perovskites Ce2MnM(Mn2Sb2)O12 with M = Mn and Zn
Source: Inorg Chem. 2025 May 16;64(21):10467–77. doi: 10.1021/acs.inorgchem.5c00653 (PMC12135034; doi:10.1021/acs.inorgchem.5c00653)
Supplement: Supplementary file 3 [file ic5c00653_si_003.pdf]

#==

data_RIETAN_publ

#==

_audit_creation_date 2025-04-01
_audit_creation_method 'Converted from *.lst using lst2cif'

#==

SUBMISSION DETAILS

_publ_contact_author_name   '?'
_publ_contact_author_address
;
;
_publ_contact_author_email   ?
_publ_contact_author_fax   '?'
_publ_contact_author_phone   '?'

_publ_requested_journal   '?'
_publ_requested_category   ?

_publ_contact_letter
;
;

#==

PROCESSING SUMMARY (IUCr Office Use Only)

#_journal_date_recd_electronic

#_journal_date_to_coeditor

#_journal_date_from_coeditor

#_journal_date_accepted

#_journal_date_printers_first

#_journal_date_printers_final

#_journal_date_proofs_out

#_journal_date_proofs_in

#_journal_coeditor_name

#_journal_coeditor_code

#_journal_coeditor_notes

#_journal_techeditor_code

#_journal_paper_category

#_journal_compatibility_tag

#_journal_techeditor_notes

#_journal_coden_ASTM

#_journal_name_full

#_journal_year

#_journal_volume

#_journal_issue

#_journal_page_first

#_journal_page_last

#_journal_suppl_publ_number

#_journal_suppl_publ_pages

#==

```
# TITLE AND AUTHOR LIST

_publ_section_title
;
;

# The loop structure below should contain the names and addresses of all
# authors, in the required order of publication. Repeat as necessary.

loop_
  _publ_author_name
  _publ_author_address
  '?'
;
;


#=======================================================================

# TEXT

_publ_section_synopsis
;
;

_publ_section_abstract
;
;

_publ_section_comment
;
```

;

_publ_section_exptl_prep
;
;

_publ_section_exptl_refinement
;
;

_publ_section_references
;
;

_publ_section_figure_captions
;
;

_publ_section_acknowledgements
;
;

#===

CRYSTAL DATA

#--
data_RIETAN_phase_1

_pd_block_id

```
  '2025-04-01|PHASE_01|..creator_name..|..instr_name..'

_pd_phase_name                     Ce2MnZnMn2Sb2O12
_cell_length_a                  7.81270(1)
_cell_length_b                  7.81270(1)
_cell_length_c                  7.94100(1)
_cell_angle_alpha                 90.0
_cell_angle_beta                  90.0
_cell_angle_gamma                 90.0
_cell_volume                    484.705(1)
_cell_formula_units               2
_symmetry_cell_setting             tetragonal
_symmetry_space_group_name_H-M        'P 42/n'
_symmetry_Int_Tables_number          86

loop_
  _symmetry_equiv_pos_site_id
  _symmetry_equiv_pos_as_xyz
    1   x,y,z
    2   -x+1/2,-y+1/2,z
    3   -y,x+1/2,z+1/2
    4   y+1/2,-x,z+1/2
    5   -x,-y,-z
    6   x+1/2,y+1/2,-z
    7   y,-x+1/2,-z+1/2
    8   -y+1/2,x,-z+1/2

loop_
  _atom_site_label
  _atom_site_symmetry_multiplicity
  _atom_site_occupancy
```

```
  _atom_site_fract_x
  _atom_site_fract_y
  _atom_site_fract_z
  _atom_site_thermal_displace_type
  _atom_site_B_iso_or_equiv
  _atom_site_type_symbol
  Ce      4 1.0       0.25       0.75       0.77499(7) Biso  0.647(10) Ce
  Mn1-SQ   2 1.0       0.25       0.25       0.75       Biso  0.63(10)  Mn
  Zn2-T    2 0.65(2)    0.75       0.75       0.75       Biso  1.00(11)  Zn
  Mn2-T    2 0.35(2)    0.75       0.75       0.75       Biso  1.00(11)  Mn
  Mn3-O    4 0.823      0.0        0.5        0.5       Biso  0.69(3)   Mn
  Zn3-O    4 0.177      0.0        0.5        0.5       Biso  0.69(3)   Zn
  Sb-O     4 1.0       0.0        0.0        0.5       Biso  0.364(11) Sb
  O1      8 1.0      -0.0532(15)  0.5701(15)  0.2327(9)  Biso  1.10(15)  O
  O2      8 1.0      -0.2291(13) -0.0480(8)   0.5889(8)  Biso  0.85(14)  O
  O3      8 1.0      -0.2591(13)  0.0701(8)  -0.0365(8)  Biso  1.28(14)  O
#----------------------------------------------------------------------
data_RIETAN_phase_2

_pd_block_id
    '2025-04-01|PHASE_02|..creator_name..|..instr_name..'

_pd_phase_name                    CeO2
_cell_length_a                  5.41102
_cell_length_b                  5.41102
_cell_length_c                  5.41102
_cell_angle_alpha                 90.0
_cell_angle_beta                  90.0
_cell_angle_gamma                 90.0
_cell_volume                    158.4300
_cell_formula_units                ?
```

```
_symmetry_cell_setting              cubic
_symmetry_space_group_name_H-M      'F m -3 m'
_symmetry_Int_Tables_number         225

loop_
  _symmetry_equiv_pos_site_id
  _symmetry_equiv_pos_as_xyz
    1  x,y,z
    2  -x,-y,z
    3  -x,y,-z
    4  x,-y,-z
    5  z,x,y
    6  z,-x,-y
    7  -z,-x,y
    8  -z,x,-y
    9  y,z,x
   10  -y,z,-x
   11  y,-z,-x
   12  -y,-z,x
   13  y,x,-z
   14  -y,-x,-z
   15  y,-x,z
   16  -y,x,z
   17  x,z,-y
   18  -x,z,y
   19  -x,-z,-y
   20  x,-z,y
   21  z,y,-x
   22  z,-y,x
   23  -z,y,x
   24  -z,-y,-x
```

25 -x,-y,-z

26 x,y,-z

27 x,-y,z

28 -x,y,z

29 -z,-x,-y

30 -z,x,y

31 z,x,-y

32 z,-x,y

33 -y,-z,-x

34 y,-z,x

35 -y,z,x

36 y,z,-x

37 -y,-x,z

38 y,x,z

39 -y,x,-z

40 y,-x,-z

41 -x,-z,y

42 x,-z,-y

43 x,z,y

44 -x,z,-y

45 -z,-y,x

46 -z,y,-x

47 z,-y,-x

48 z,y,x

49 x+1/2,y+1/2,z

50 -x+1/2,-y+1/2,z

51 -x+1/2,y+1/2,-z

52 x+1/2,-y+1/2,-z

53 z+1/2,x+1/2,y

54 z+1/2,-x+1/2,-y

55 -z+1/2,-x+1/2,y

56 -z+1/2,x+1/2,-y

57 y+1/2,z+1/2,x

58 -y+1/2,z+1/2,-x

59 y+1/2,-z+1/2,-x

60 -y+1/2,-z+1/2,x

61 y+1/2,x+1/2,-z

62 -y+1/2,-x+1/2,-z

63 y+1/2,-x+1/2,z

64 -y+1/2,x+1/2,z

65 x+1/2,z+1/2,-y

66 -x+1/2,z+1/2,y

67 -x+1/2,-z+1/2,-y

68 x+1/2,-z+1/2,y

69 z+1/2,y+1/2,-x

70 z+1/2,-y+1/2,x

71 -z+1/2,y+1/2,x

72 -z+1/2,-y+1/2,-x

73 -x+1/2,-y+1/2,-z

74 x+1/2,y+1/2,-z

75 x+1/2,-y+1/2,z

76 -x+1/2,y+1/2,z

77 -z+1/2,-x+1/2,-y

78 -z+1/2,x+1/2,y

79 z+1/2,x+1/2,-y

80 z+1/2,-x+1/2,y

81 -y+1/2,-z+1/2,-x

82 y+1/2,-z+1/2,x

83 -y+1/2,z+1/2,x

84 y+1/2,z+1/2,-x

85 -y+1/2,-x+1/2,z

86 y+1/2,x+1/2,z

87 -y+1/2,x+1/2,-z

88 y+1/2,-x+1/2,-z

89 -x+1/2,-z+1/2,y

90 x+1/2,-z+1/2,-y

91 x+1/2,z+1/2,y

92 -x+1/2,z+1/2,-y

93 -z+1/2,-y+1/2,x

94 -z+1/2,y+1/2,-x

95 z+1/2,-y+1/2,-x

96 z+1/2,y+1/2,x

97 x+1/2,y,z+1/2

98 -x+1/2,-y,z+1/2

99 -x+1/2,y,-z+1/2

100 x+1/2,-y,-z+1/2

101 z+1/2,x,y+1/2

102 z+1/2,-x,-y+1/2

103 -z+1/2,-x,y+1/2

104 -z+1/2,x,-y+1/2

105 y+1/2,z,x+1/2

106 -y+1/2,z,-x+1/2

107 y+1/2,-z,-x+1/2

108 -y+1/2,-z,x+1/2

109 y+1/2,x,-z+1/2

110 -y+1/2,-x,-z+1/2

111 y+1/2,-x,z+1/2

112 -y+1/2,x,z+1/2

113 x+1/2,z,-y+1/2

114 -x+1/2,z,y+1/2

115 -x+1/2,-z,-y+1/2

116 x+1/2,-z,y+1/2

117 z+1/2,y,-x+1/2

118 z+1/2,-y,x+1/2

119 -z+1/2,y,x+1/2

120 -z+1/2,-y,-x+1/2

121 -x+1/2,-y,-z+1/2

122 x+1/2,y,-z+1/2

123 x+1/2,-y,z+1/2

124 -x+1/2,y,z+1/2

125 -z+1/2,-x,-y+1/2

126 -z+1/2,x,y+1/2

127 z+1/2,x,-y+1/2

128 z+1/2,-x,y+1/2

129 -y+1/2,-z,-x+1/2

130 y+1/2,-z,x+1/2

131 -y+1/2,z,x+1/2

132 y+1/2,z,-x+1/2

133 -y+1/2,-x,z+1/2

134 y+1/2,x,z+1/2

135 -y+1/2,x,-z+1/2

136 y+1/2,-x,-z+1/2

137 -x+1/2,-z,y+1/2

138 x+1/2,-z,-y+1/2

139 x+1/2,z,y+1/2

140 -x+1/2,z,-y+1/2

141 -z+1/2,-y,x+1/2

142 -z+1/2,y,-x+1/2

143 z+1/2,-y,-x+1/2

144 z+1/2,y,x+1/2

145 x,y+1/2,z+1/2

146 -x,-y+1/2,z+1/2

147 -x,y+1/2,-z+1/2

148 x,-y+1/2,-z+1/2

149 z,x+1/2,y+1/2

150 z,-x+1/2,-y+1/2

151 -z,-x+1/2,y+1/2

152 -z,x+1/2,-y+1/2

153 y,z+1/2,x+1/2

154 -y,z+1/2,-x+1/2

155 y,-z+1/2,-x+1/2

156 -y,-z+1/2,x+1/2

157 y,x+1/2,-z+1/2

158 -y,-x+1/2,-z+1/2

159 y,-x+1/2,z+1/2

160 -y,x+1/2,z+1/2

161 x,z+1/2,-y+1/2

162 -x,z+1/2,y+1/2

163 -x,-z+1/2,-y+1/2

164 x,-z+1/2,y+1/2

165 z,y+1/2,-x+1/2

166 z,-y+1/2,x+1/2

167 -z,y+1/2,x+1/2

168 -z,-y+1/2,-x+1/2

169 -x,-y+1/2,-z+1/2

170 x,y+1/2,-z+1/2

171 x,-y+1/2,z+1/2

172 -x,y+1/2,z+1/2

173 -z,-x+1/2,-y+1/2

174 -z,x+1/2,y+1/2

175 z,x+1/2,-y+1/2

176 z,-x+1/2,y+1/2

177 -y,-z+1/2,-x+1/2

178 y,-z+1/2,x+1/2

179 -y,z+1/2,x+1/2

```
  180  y,z+1/2,-x+1/2
  181  -y,-x+1/2,z+1/2
  182  y,x+1/2,z+1/2
  183  -y,x+1/2,-z+1/2
  184  y,-x+1/2,-z+1/2
  185  -x,-z+1/2,y+1/2
  186  x,-z+1/2,-y+1/2
  187  x,z+1/2,y+1/2
  188  -x,z+1/2,-y+1/2
  189  -z,-y+1/2,x+1/2
  190  -z,y+1/2,-x+1/2
  191  z,-y+1/2,-x+1/2
  192  z,y+1/2,x+1/2

loop_
  _atom_site_label
  _atom_site_symmetry_multiplicity
  _atom_site_occupancy
  _atom_site_fract_x
  _atom_site_fract_y
  _atom_site_fract_z
  _atom_site_thermal_displace_type
  _atom_site_B_iso_or_equiv
  _atom_site_type_symbol
  Ce51     4 1.0       0.0        0.0        0.0       Biso  0.5       Ce
  O511     8 1.0       0.25       0.25       0.25      Biso  1.0       O
#----------------------------------------------------------------------
data_RIETAN_phase_3

_pd_block_id
    '2025-04-01|PHASE_03|..creator_name..|..instr_name..'
```

```
_pd_phase_name                    Na5Co15.5Te6O36-related
_cell_length_a                    9.62291
_cell_length_b                    9.62291
_cell_length_c                    9.35094
_cell_angle_alpha                 90.0
_cell_angle_beta                  90.0
_cell_angle_gamma                 120.0
_cell_volume                      749.8920
_cell_formula_units               ?
_symmetry_cell_setting            hexagonal
_symmetry_space_group_name_H-M    'P 63/m'
_symmetry_Int_Tables_number       176

loop_
  _symmetry_equiv_pos_site_id
  _symmetry_equiv_pos_as_xyz
    1   x,y,z
    2   -y,x-y,z
    3   -x+y,-x,z
    4   -x,-y,z+1/2
    5   y,-x+y,z+1/2
    6   x-y,x,z+1/2
    7   -x,-y,-z
    8   y,-x+y,-z
    9   x-y,x,-z
   10   x,y,-z+1/2
   11   -y,x-y,-z+1/2
   12   -x+y,-x,-z+1/2

loop_
```

```
  _atom_site_label
  _atom_site_symmetry_multiplicity
  _atom_site_occupancy
  _atom_site_fract_x
  _atom_site_fract_y
  _atom_site_fract_z
  _atom_site_thermal_displace_type
  _atom_site_B_iso_or_equiv
  _atom_site_type_symbol
  O1f     6 1.0       0.8841     0.1746     0.25      Biso  0.5      O
  O2f    12 1.0       0.61       0.1341     0.1003    Biso  0.5      O
  O3f    12 1.0       0.6866    -0.1078     0.0927    Biso  0.5      O
  O4f     6 1.0       0.4165    -0.1311     0.25      Biso  0.5      O
  Na1f    4 0.9717    0.0        0.0       -0.344     Biso  0.5      Mn
  Co1f   12 1.1782    0.34381   -0.0066     0.08778   Biso  0.5      Mn
  Te1f    6 1.1388    0.65608    0.00984    0.25      Biso  0.5      Sb
  Co2f    2 1.1021    0.6667     0.3333     0.25      Biso  0.5      Mn
  Co3f    4 1.7429    0.3333    -0.3333     0.0819    Biso  0.5      Mn
#----------------------------------------------------------------------
data_RIETAN_phase_4

_pd_block_id
    '2025-04-01|PHASE_04|..creator_name..|..instr_name..'

_pd_phase_name                     La3Mn2Sb3O14 R-3m
_cell_length_a                  7.44282
_cell_length_b                  7.44282
_cell_length_c                  17.62570
_cell_angle_alpha                 90.0
_cell_angle_beta                 90.0
_cell_angle_gamma                 120.0
```

```
_cell_volume                    845.5749
_cell_formula_units             ?
_symmetry_cell_setting          trigonal
_symmetry_space_group_name_H-M  'R -3 m'
_symmetry_Int_Tables_number     166

loop_
  _symmetry_equiv_pos_site_id
  _symmetry_equiv_pos_as_xyz
   1  x,y,z
   2  -y,x-y,z
   3  -x+y,-x,z
   4  y,x,-z
   5  x-y,-y,-z
   6  -x,-x+y,-z
   7  -x,-y,-z
   8  y,-x+y,-z
   9  x-y,x,-z
   10  -y,-x,z
   11  -x+y,y,z
   12  x,x-y,z
   13  x+2/3,y+1/3,z+1/3
   14  -y+2/3,x-y+1/3,z+1/3
   15  -x+y+2/3,-x+1/3,z+1/3
   16  y+2/3,x+1/3,-z+1/3
   17  x-y+2/3,-y+1/3,-z+1/3
   18  -x+2/3,-x+y+1/3,-z+1/3
   19  -x+2/3,-y+1/3,-z+1/3
   20  y+2/3,-x+y+1/3,-z+1/3
   21  x-y+2/3,x+1/3,-z+1/3
   22  -y+2/3,-x+1/3,z+1/3
```

```
  23  -x+y+2/3,y+1/3,z+1/3
  24  x+2/3,x-y+1/3,z+1/3
  25  x+1/3,y+2/3,z+2/3
  26  -y+1/3,x-y+2/3,z+2/3
  27  -x+y+1/3,-x+2/3,z+2/3
  28  y+1/3,x+2/3,-z+2/3
  29  x-y+1/3,-y+2/3,-z+2/3
  30  -x+1/3,-x+y+2/3,-z+2/3
  31  -x+1/3,-y+2/3,-z+2/3
  32  y+1/3,-x+y+2/3,-z+2/3
  33  x-y+1/3,x+2/3,-z+2/3
  34  -y+1/3,-x+2/3,z+2/3
  35  -x+y+1/3,y+2/3,z+2/3
  36  x+1/3,x-y+2/3,z+2/3

loop_
  _atom_site_label
  _atom_site_symmetry_multiplicity
  _atom_site_occupancy
  _atom_site_fract_x
  _atom_site_fract_y
  _atom_site_fract_z
  _atom_site_thermal_displace_type
  _atom_site_B_iso_or_equiv
  _atom_site_type_symbol
  Mn-1a    3 0.98      0.0        0.0        0.5       Biso  0.5      Mn
  Ce-2a    9 0.82      0.5        0.0        0.5       Biso  0.5      Ce
  Mn-2a    3 0.87      0.0        0.0        0.0       Biso  0.5      Mn
  Sb-2a    9 1.0       0.5        0.0        0.0       Biso  1.9      Sb
  O-1n    18 1.0       0.4762     0.5238     0.1116    Biso  0.8      O
  O-2n    18 1.0       0.4664     0.5336     0.3498    Biso  0.8      O
```

```
  O-3n    6 1.0      0.0       0.0       0.114     Biso  0.8      O

#==============================================================================

# POWDER SPECIMEN AND EXPERIMENTAL DATA

data_RIETAN_p_01

_pd_block_id
     '2025-04-01|POWSET_01|..creator_name..|..instr_name..'
_pd_meas_datetime_initialed           ?
_pd_meas_info_author_name             "?"
_pd_meas_info_author_email            ?
_pd_meas_info_author_address
;
;

_pd_calc_method                   "Rietveld Refinement"
_diffrn_ambient_temperature          ?
_diffrn_ambient_environment          ?
_diffrn_source                    '?'
_diffrn_source_target               ?
_diffrn_source_type                 ?
_diffrn_measurement_device_type      '?'
_diffrn_detector                  '?'
_diffrn_detector_type               ?
_pd_meas_scan_method                step
_pd_meas_special_details
;
;
_diffrn_radiation_type              'synchrotron X-ray'
```

_diffrn_radiation_wavelength 0.61974

_diffrn_radiation_monochromator none

_pd_meas_2theta_range_min 4.002

_pd_meas_2theta_range_max 71.250

_pd_meas_2theta_range_inc 0.006

_pd_meas_number_of_points 11209

#==

REFINEMENT DATA

_pd_proc_ls_special_details

;

;

_pd_proc_ls_profile_function ?

_pd_proc_ls_background_function '?'

_pd_proc_ls_pref_orient_corr

;

;

_pd_proc_ls_prof_R_factor 0.0688

_pd_proc_ls_prof_wR_factor 0.0908

_pd_proc_ls_prof_wR_expected 0.0167

_refine_special_details

;

;

_refine_ls_structure_factor_coef Inet

_refine_ls_matrix_type ?

_refine_ls_weighting_sheme '?'

_refine_ls_hydrogen_treatment noref

_refine_ls_extinction_method none

_refine_ls_extinction_coef ?

_refine_ls_number_parameters ?

_refine_ls_number_constraints 35

_refine_ls_goodness_of_fit_all 5.44

#--eof--eof--eof--eof--eof--eof--eof--eof--eof--eof--eof--eof--eof--eof--eof--#
